# The impact of different diagnostic criteria on the association of sarcopenia with injurious falls in the CLSA

**DOI:** 10.1002/jcsm.12622

**Published:** 2020-09-17

**Authors:** Alexandra J. Mayhew, Stuart M. Phillips, Nazmul Sohel, Lehana Thabane, Paul D. McNicholas, Russell J. de Souza, Gianni Parise, Parminder Raina

**Affiliations:** ^1^ Department of Health Research Methods, Evidence, and Impact McMaster University Hamilton Ontario Canada; ^2^ Labarge Centre for Mobility in Aging McMaster University Hamilton Ontario Canada; ^3^ McMaster Institute for Research on Aging McMaster University Hamilton Ontario Canada; ^4^ Department of Kinesiology McMaster University Hamilton Ontario Canada; ^5^ Biostatistics Unit, Research Institute at St Joes St. Joseph's Healthcare Hamilton Hamilton Ontario L8N 4A6 Canada; ^6^ Department of Mathematics & Statistics McMaster University Hamilton Ontario Canada; ^7^ Population Genomics Program, Chanchlani Research Centre McMaster University Hamilton Ontario Canada

**Keywords:** Sarcopenia, Falls, Muscle mass, Muscle strength, Muscle function, CLSA

## Abstract

**Background:**

Sarcopenia definitions recommend different combinations of variables (lean mass, strength, and physical function) and different methods of adjusting lean mass. The purpose of this paper was to address the gaps in the literature regarding how differences in the operationalization of sarcopenia impact the association between sarcopenia and injurious falls.

**Methods:**

Participants included 9936 individuals from the Canadian Longitudinal Study on Aging aged ≥65 years at baseline (2012–2015), with complete data for sarcopenia‐related variables, injurious falls, and covariates. Sarcopenia was defined using all combinations of muscle variables (lean mass, grip strength, chair rise test, and gait speed) and methods of adjusting lean mass (height^2^, weight, body mass index (BMI), and regressing on height and fat mass) recommended by the expert group sarcopenia definitions. Multiple cut off values for the measures were explored. The association between sarcopenia and injurious falls (0, 1, or 2+ falls) measured 18 months after baseline data collection were assessed using proportional odds regression models.

**Results:**

In men (*n* = 5162, 72.9 ± 5.6 years), the odds of having a higher level of injurious falls was between 1.43 and 2.14 greater when sarcopenia was defined as (i) lean mass adjusted for weight only; (ii) grip strength (<30 or <26 kg) only; (iii) lean mass adjusted for weight and grip strength (<30 or <26 kg); (iv) lean mass adjusted for BMI and grip strength (<26 kg); and (v) lean mass adjusted using the regression technique and grip strength (<30 or <26 kg). In women (*n* = 4774, 72.8 ± 5.6 years), only the combination of lean mass adjusted using regression with gait speed (<0.8 m/s) was associated with a significantly higher odds (1.46, 95% confidence interval: 1.01–2.10, *P* = 0.04) of having a higher level of injurious falls.

**Conclusions:**

Sarcopenia definitions based on different combinations of muscle variables and methods of adjusting lean mass are not equally associated with injurious falls. In men, definitions including grip strength but not gait speed or the chair rise test, and adjusting lean mass for weight, BMI, or using the residual technique but not height^2^, tended to be associated with injurious falls. In women, sarcopenia was generally not associated with injurious falls regardless of the definition used.

## Introduction

Sarcopenia is now recognized as a muscle disorder not only in older adults, but strongly associated with advanced age. The deficits in muscle mass approximated using appendicular lean mass (ALM) measured by dual‐energy X‐ray absorptiometry (DXA) or bioelectrical impedance analysis, muscle strength, and physical function that characterize sarcopenia are thought to be associated with clinically relevant outcomes. Falls are one of the outcomes of greatest interest for sarcopenia due the biological link between muscle strength and physical function with falls.^1^ Falls, often defined as ‘inadvertently coming to rest on the ground, floor or another lower level’ are a serious health problem in older adults.^2^ Approximately one third of adults ≥65 years fall annually,^3^ and 6% experience a fall‐related injury.^4^ These fall‐related injuries such as hip fractures, traumatic brain injuries, and upper limb injuries, accounted for more than 80% of injury‐related hospitalizations in that age group and are a significant cost to health care systems.^4^ There are many identified risk factors for falls, which can broadly be classified as biological, behavioural, socio‐economic, and environmental.^2^ Multifactorial falls risk assessments have routinely been identified as the first step in preventing falls.^5^ However, it is unclear if sarcopenia is a useful biological measure for identifying potential fallers.

The results of previous studies investigating the association of sarcopenia with falls have been inconclusive with approximately half of the studies finding a significant association.^6^ However, there are substantial differences between studies, including how sarcopenia was operationalized. Sarcopenia can be identified as low lean mass only or as low lean mass combined with low muscle strength or low physical function.^7^, ^8^, ^9^, ^10^, ^11^, ^12^ Different adjustment techniques for low lean mass and cut offs for the variables are also recommended.^7^, ^8^, ^9^, ^10^, ^11^, ^12^ Depending on which definition is used, sarcopenia prevalence is between 9.9% and 40.4% of community‐dwelling older adults^13^ with evidence of limited agreement between definitions.^14^, ^15^, ^16^, ^17^, ^18^, ^19^ Three studies compared different sarcopenia definitions within the same population and found that definitions including low lean mass combined with muscle strength and/or physical function were typically associated with falls, but not definitions including just lean mass.^20^, ^21^, ^22^ However, these studies are limited by only considering a subset of sarcopenia definitions, and it remains unclear if there are specificv sarcopenia definitions that are most appropriate for identifying people that may fall.

Determining which, if any, sarcopenia definitions are most strongly associated with falls serves two key purposes. First, the sarcopenia research community is working to identify a unified sarcopenia definition. Determining which definitions are most strongly associated with health outcomes such as falls is a critical step in this process. Second, knowing the best method of operationalizing sarcopenia in the context of falls may help inform falls prevention strategies. The objective of this study was to assess the impact of different methods of operationalizing sarcopenia on the strength of the association between sarcopenia and injurious falls.

## Methods

### Setting and study population

The Canadian Longitudinal Study on Aging (CLSA) is a national, longitudinal research platform including 51 338 participants aged 45–85 years at baseline from the 10 Canadian provinces. To be eligible for the study, participants had to be physically and cognitively able to participate on their own and not living in institutions such as long‐term care. Participants were recruited in the tracking cohort (*n* = 21 241) and the comprehensive cohort (*n* = 30 097). Tracking cohort participants were randomly selected from the 10 provinces and completed interviews by phone. Participants in the comprehensive cohort were randomly selected from within 25–50 km of 11 data collection sites, which are located in seven provinces. In addition to being interviewed in‐person, comprehensive cohort participants completed in‐depth physical assessments and provided blood and urine samples. Details on the study design have been described elsewhere.^23^ The sample for this study was limited to participants aged ≥65 years. Only participants from the comprehensive cohort were included because the required physical assessment data was not collected in tracking cohort participants. This project used data collected during baseline (September 2011 to May 2015) as well as data collected during a maintaining contact questionnaire administered approximately 18 months after baseline data collection.

### Clinical measurements

All data were collected by trained research assistants. Height was measured using a stadiometer. The mean value of two measurements was used for analyses. Weight was measured using a digital scale. Body mass index (BMI) was calculated as weight in kilograms divided by height squared. Lean mass was measured by DXA using Hologic Discovery A™. The DXA machine was calibrated daily using a spine phantom, weekly using a whole body step phantom, and yearly using a gold standard phantom. Hand grip strength was measured using the JTech Tracker Freedom® Wireless Grip Dynamometer. Three repetitions were performed with the dominant hand, the highest of which was used in the analyses. Grip strength measured using a dynamometer has excellent reliability and is predictive of falls, disability, and impaired health‐related quality of life.^24^ Gait speed was measured using a 4 m walk course at normal walking speed. This test has excellent test–retest reliability and is significantly associated with self‐rated health and performance on chair rise and balance tests.^25^, ^26^ The chair rise test was conducted by asking participants to rise to a fully standing position from seated five times as quickly as possible.^27^

### Injurious falls assessment

Injurious falls were assessed during the maintaining contact interview appropriately 18 months after participants visited the data collection site. Participants were asked if they had experienced a fall where they were hurt enough to limit some of their normal activities in the past 12 months, and if they had a fall, how many times they had fallen. Participants were categorized as having not fallen, fallen once, or fallen two or more times.

### Sarcopenia operationalization

Sarcopenia was defined based on the recommendations of the expert‐group definitions.^9^, ^10^, ^11^ Lean mass measured by ALM was adjusted by height^2^, weight, BMI, and regressing ALM on fat mass and height. Muscle strength was measured using grip strength with cut offs of 30, 27, and 26 kg for men and 20 and 16 kg for women, as well as the chair rise test with a cut off of ≥15 s. Physical function was measured using gait speed using cut offs of 0.8 and 1.0 m/s.^9^, ^10^, ^11^, ^12^ Cut offs corresponding to the lowest sex‐specific 10th, 20th, and 40th percentiles of ALM values for each adjustment technique were used (*Table* 1). Each combination of muscle variables (lean mass alone, lean mass and muscle strength, and lean mass and physical function), methods of adjusting lean mass, and cut offs were used to define sarcopenia. Definitions including a grip strength cut off of 27 kg for men, the 1.0 m/s gait speed cut off, lean muscle mass thresholds corresponding to the 10th and 40th percentiles, and those including the chair rise were included as secondary analyses.

**TABLE 1:** Low lean mass cut offs

Method of adjusting lean mass	Percentile	Low lean mass cut offs	
		Men	Women
ALM/height^2^	10th	6.83	5.21
	20th	7.19	5.56
	40th	7.72	6.03
ALM/weight	10th	0.25	0.20
	20th	0.27	0.21
	40th	0.28	0.22
ALM/BMI	10th	0.74	0.49
	20th	0.78	0.52
	40th	0.85	0.57
ALM residuals^a^, ^b^	10th	−3.41	−2.30
	20th	−2.32	−1.58
	40th	−0.78	−0.60

ALM, appendicular lean mass; BMI, body mass index.

^a^ Regression equation in male patients: Predicted ALM = −23.8 + 25.2 (height in m) + 0.17 (fat mass in kg).

^b^ Regression equation in male patients: Predicted ALM = −16.1 + 16.9 (height in m) + 0.18 (fat mass in kg).

### Statistical analyses

Of the 30 097 participants at baseline, 12 628 were ≥65 years. Participants missing data for sarcopenia (*n* = 1818), injurious falls (*n* = 459), and those missing covariate data (*n* = 415) were excluded, leaving 9936 participants for analyses. The CLSA provides inflation weights and analytical weights, which were used for prevalence estimates and regression modelling respectively, that allow the results to reflect the population of Canada.^28^ All statistical analyses were completed using SAS (Version 12.3).

A proportional odds model (SAS procgenmod)^29^ was used to estimate the odds ratios and 95% confidence intervals (CIs) for the outcome of injurious falls categorized as 0, 1, or 2+ falls in the previous year. The proportional odds model takes the ordinal nature of the falls data into consideration. Based on the proportionality assumption, the odds ratio of having 1+ injurious falls vs. 0 falls is the same as the odds of having 2+ injurious falls vs. ≤1 fall for each explanatory variable. Therefore, a single odds ratio is estimated for each explanatory variable, which is interpreted as the odds of having reported at least one additional injurious fall. There are two *y*‐intercepts, one for the outcome category of having 1+ falls vs. 0 falls and the other for having 2+ falls vs. ≤1 fall. The proportionality assumption was tested and was not found to be violated for the sarcopenia variables with falls before or after adjustment with other covariates.

Potential covariates were identified in the literature based on their relevance to falls and sarcopenia.^30^ The univariate association between each variable and injurious falls was assessed; variables with a Wald statistic *P* value of ≤0.25 were considered candidates for the model. Age was automatically included in the model, and other potential covariates were added in one at a time based on statistical significance. Variables for which the deviance statistic was statistically significant (*χ* ^2^ test *P* value of <0.05) or those which impacted the strength of the association between sarcopenia and injurious falls were kept in the model.^31^ The final model included age (65–74 and ≥75 years), urinary incontinence, the use of mobility devices, general health (fair or poor vs. excellent, very good, or good), and the presence of pain or discomfort for which the deviance was significant reduced (*P* < 0.05) as well as diabetes and osteoarthritis, which had *P* values of <0.10 and impacted the strength of the association between sarcopenia and injurious falls. The covariates identified for inclusion in the model were the same regardless of the sarcopenia definition used. Analyses were stratified by sex. The assumption of proportionality was violated for general health and osteoarthritis in men, and urinary incontinence in women. Therefore, a partial proportional odds model (SAS proclogistic), which cannot accommodate weights, was included as a sensitivity analyses to allow for covariates violating the proportional odds assumption to have two separate odds ratios estimated.

The discriminative ability of each sarcopenia definition for the outcomes of 1+ and 2+ injurious falls were assessed using area under the receiver operator curve (AUC) analyses. The direction of the misclassification was assessed by calculating sensitivity and specificity. These analyses were unadjusted in order to estimate the discriminative ability of sarcopenia for injurious falls without consideration of other variables such as would be the case in clinical settings.

## Results

### Participant characteristics

The mean age of the participants was 72.9 ± 5.6 years, and 52.0% of the sample were men (*Table* 2). Men had greater ALM (24.4 ± 3.7 kg) compared with women (16.3 ± 2.9 kg), faster gait speed (0.94 ± 0.19 m/s vs. 0.91 ± 0.19 m/s), and greater grip strength (39.5 ± 8.5 kg vs. 23.9 ± 5.5 kg). Injurious falls were more common in women than in men with 13.3% of women and 9.5% of men reporting ≥1 injurious falls in the previous 12 months. Weighted characteristics are available in supporting information *Table* S1. In comparison with those with complete data, individuals with missing data (≤10% for each variable) were approximately 1 year older, had lower lean mass, gait speed, and grip strength, experienced more injurious falls, and were generally in poorer health (*Table* S2).

**TABLE 2:** Participant characteristics

Characteristic	Men (*n* = 5162)		Women (*N* = 4774)	
	Mean or *N*	SD or %	Mean or *N*	SD or %
Age (years)	72.9	5.55	72.8	5.64
European (%)	4951	95.9	4651	97.4
Height (cm)	173.9	6.74	159.9	6.33
Weight (kg)	84.4	14.07	70.8	14.29
BMI (kg/m^2^)	27.9	4.21	27.7	5.45
Total body fat mass (%)	25.3	8.03	29.5	9.36
Appendicular lean mass (kg)	24.4	3.68	16.3	2.87
ALM/height^2^	8.1	1.02	6.4	1.01
ALM/weight	29.1	2.97	23.3	2.73
ALM/BMI	0.9	0.12	0.6	0.09
ALM residuals	0.00	2.78	0.00	1.95
Gait speed (m/s)	0.94	0.19	0.91	0.19
Grip strength (kg)	39.5	8.52	23.9	5.54
Chair rise test (s)	13.9	3.76	14.5	4.62
Number of injurious falls in previous year (%)				
Zero	4683	90.5	4161	86.7
One	375	7.5	468	10.1
Two or more	104	2.0	145	3.2
Self‐rated general health (%)				
Fair or poor	404	7.5	371	8.0
Good, very good, or excellent	4758	92.5	4403	92.0
Presence of pain or discomfort (%)	1631	32.5	2023	43.0
Self‐rated hearing (%)				
Fair or poor	912	16.9	521	11.1
Good, very good, or excellent	4250	83.1	4252	88.9
Urinary incontinence (%)	400	7.3	690	14.8
Household income (%)				
<$20,000	158	2.9	387	8.9
≥$20,000 to <$50,000	1239	26.9	1765	40.5
≥$50,000 to <$100,000	2164	43.6	1497	35.5
≥$100,000 to <$150,000	842	16.9	415	10.5
≥$150,000	462	9.6	182	4.5
Smoking status (%)				
Current	262	5.1	231	4.9
Never or former	4861	94.9	4500	95.1
COPD (%)	319	5.8	372	7.3
Depression (%)	523	9.7	848	17.4
Neurological conditions (%)	349	7.1	808	16.8
Arthritis (%)	1371	27.0	2019	41.4
Diabetes (%)	1243	24.1	848	17.4
Stroke (%)	379	6.8	303	6.3
Osteoporosis (%)	186	3.7	1164	24.7

ALM, appendicular lean mass; BMI, body mass index; COPD, chronic obstructive pulmonary disease.

### Sarcopenia—primary analyses

In men, using the 20th percentile cut offs for lean mass, the single variables that were significantly associated with having a greater odds of a higher level of injurious falls were ALM adjusted for weight (1.43, 95% CI: 1.07–1.90), and grip strength using the cut offs of 30 kg (1.43, 95% CI: 1.02–1.99) and 26 kg (1.78, 95% CI: 1.12–2.82) (*Table* 3). The combinations of variables that were significantly associated with a greater odds of a higher level of injurious falls were ALM adjusted for weight and using the residual technique in combination with low grip strength (30 or 26 kg) with odds ratios of between 1.64 and 2.14. Lean mass adjusted for BMI with a grip strength of <26 kg was associated with a 1.97 (95% CI: 1.06–3.63) greater odds of a higher level of injurious falls. Regardless of the definition of sarcopenia, the AUC values ranged from 0.50 to 0.54 for having at least one injurious fall and 0.51 to 0.59 for having two or more injurious falls (*Table* S3).

**TABLE 3:** Association between sarcopenia and falls in women using different methods of operationalizing sarcopenia

Sarcopenia definition		Number of participants with sarcopenia (%)	*y*‐Intercept for 1+ injurious falls vs. no falls	*y*‐Intercept for 2+ injurious falls vs. zero or one falls	Odds of falling	95% Confidence interval	*P* value
Combination of muscle variables	Method of adjusting lean mass						
Combination of muscle variables	Method of adjusting lean mass
Lean mass only (20th percentile)	Height^2^ ^a^	960 (20.1)	−2.28	−3.89	1.07	0.83–1.37	0.619
	Weight^b^	936 (19.6)	−2.26	−3.86	0.93	0.72–1.19	0.551
	BMI^c^	930 (19.5)	−2.27	−3.87	1.00	0.78–1.28	0.997
	Residuals^d^	950 (19.9)	−2.28	−3.88	1.06	0.83–1.36	0.618
Grip strength only <20 kg		1092 (22.9)	kg−2.27	−3.87	1.01	0.79–1.29	0.948
Grip strength only <16 kg		330 (6.9)	−2.27	−3.87	0.89	0.60–1.34	0.584
Gait speed only <0.8 m/s		1394 (29.2)	−2.28	−3.89	1.14	0.90–1.43	0.279
Lean mass (20th percentile) and grip strength <20 kg	Height^2^	329 (6.9)	−2.27	−3.87	1.09	0.74–1.60	0.670
	Weight	300 (6.3)	−2.27	−3.87	0.56	0.35–0.90	0.016*
	BMI	361 (7.6)	−2.27	−3.87	0.80	0.54–1.18	0.252
	Residuals	280 (5.9)	−2.27	−3.87	1.00	0.66–1.52	0.998
Lean mass (20th percentile) and grip strength <16 kg	Height^2^	118 (2.5)	−2.27	−3.87	0.95	0.51–1.77	0.865
	Weight	85 (1.8)	−2.27	−3.87	0.48	0.20–1.20	0.116
	BMI	121 (2.5)	−2.27	−3.87	0.72	0.37–1.40	0.333
	Residuals	86 (1.8)	−2.27	−3.87	0.68	0.29–1.55	0.357
Lean mass (20th percentile) and gait speed <0.8 m/s	Height^2^	265 (5.6)	−2.28	−3.88	1.29	0.86–1.94	0.220
	Weight	409 (8.6)	−2.27	−3.87	0.97	0.69–1.38	0.878
	BMI	393 (8.2)	−2.27	−3.87	1.09	0.77–1.54	0.629
	Residuals	304 (6.4)	−2.28	−3.88	1.46	1.01–2.10	0.043*

BMI, body mass index.

* Statistically significant (*P* < 0.05).

^a^ Cut off value of 5.56 kg/m^2^ (lean mass/height in metres).

^b^ Cut off value of 0.21 (lean mass/total mass).

^c^ Cut off value of 0.52 (lean mass/body mass index).

^d^ Cut off value of −1.58 (residual of lean mass regressed on fat mass and height).

In our analyses, there are two thresholds used to define the outcome of falls. The first compares those with 1+ injurious falls to those with 0 falls and the second compares those with 2+ injurious falls to those with ≤1 fall. The odds ratio for each covariate is the same regardless of the threshold for the outcome. The *y*‐intercepts for having 1+ falls vs. 0 falls were between −2.68 and −2.72 and between −4.30 and −4.34 for having 2+ falls vs. ≤1 fall. For each threshold, the *y*‐intercept represents the odds of the outcome occurring when all covariate values are 0. To interpret the *y*‐intercept values, the natural exponent of each *y*‐intercept is calculated. For example using the *y*‐intercepts of −2.68 and −4.30, the odds of having 1+ falls vs. 0 falls is 0.069 (*e*^−2.68^) and the odds of having 2+ falls vs. ≤1 fall is 0.014 (*e*^−4.30^). Therefore, the odds of having 1+ injurious falls is approximately five times higher (0.069/0.014 = 4.93) than the odds of having 2+ injurious falls. Although these values refer to a person with all covariate values equalling zero, the ratio of the odds for the *y*‐intercepts remain the same regardless of the value of the covariates (*Table* 4).

**TABLE 4:** Association between sarcopenia and injurious falls in men using different methods of operationalizing sarcopenia

Sarcopenia definition		Number of participants with sarcopenia (%)	*y*‐Intercept for 1+ injurious falls vs. no falls	*y*‐Intercept for 2+ injurious falls vs. zero or one falls	Odds of falling	95% Confidence interval	*P* value
Combination of muscle variables	Method of adjusting lean mass^a^						
Lean mass only (20th percentile)	Height^2^ ^a^	1016 (19.7)	−2.71	−4.33	1.18	0.88–1.59	0.276
	Weight^b^	995 (19.3)	−2.72	−4.34	1.43	1.07–1.90	0.016*
	BMI^c^	994 (19.3)	−2.69	−4.31	1.06	0.78–1.44	0.703
	Residuals^d^	1015 (19.7)	−2.72	−4.34	1.30	0.97–1.73	0.080
Grip strength only <30 kg		668 (12.9)	−2.70	−4.32	1.43	1.02–1.99	0.035*
Grip strength only <26 kg		257 (5.0)	−2.69	−4.31	1.78	1.12–2.82	0.014*
Gait speed only <0.8 m/s		1138 (22)	−2.71	−4.33	1.30	0.98–1.73	0.073
Lean mass (20th percentile) and grip strength <30 kg	Height^2^	244 (4.7)	−2.68	−4.30	1.15	0.67–1.97	0.615
	Weight	232 (4.5)	−2.68	−4.30	1.64	1.01–2.66	0.048*
	BMI	294 (5.7)	−2.69	−4.31	1.51	0.96–2.36	0.073
	Residuals	215 (4.2)	−2.69	−4.30	1.66	1.00–2.75	0.049*
Lean mass (20th percentile) and grip strength <26 kg	Height^2^	106 (2.1)	−2.68	−4.30	1.18	0.54–2.60	0.677
	Weight	105 (2.0)	−2.68	−4.30	2.14	1.12–4.09	0.021*
	BMI	123 (2.4)	−2.69	−4.31	1.97	1.06–3.63	0.031*
	Residuals	90 (1.7)	−2.68	−4.30	2.07	1.03–4.15	0.040*
Lean mass (20th percentile) and gait speed <0.8 m/s	Height^2^	254 (4.9)	−2.68	−4.30	1.10	0.65–1.86	0.732
	Weight	348 (6.7)	−2.68	−4.30	1.27	0.82–1.96	0.283
	BMI	363 (7.0)	−2.68	−4.30	1.01	0.65–1.59	0.961
	Residuals	280 (5.4)	−2.68	−4.30	1.30	0.81–2.10	0.276

BMI, body mass index.

* Statistically significant (*P* < 0.05).

^a^ Cut off value of 7.19 kg/m^2^ (lean mass/height in metres).

^b^ Cut off value of 0.27 (lean mass/total mass).

^c^ Cut off value of 0.78 (lean mass/body mass index).

^d^ Cut off value of −2.32 (residual of lean mass regressed on fat mass and height).

In women, ALM adjusted using the regression technique with a gait speed of <0.8 m/s was the only definition associated with an increased odds of injurious falls (1.46, 95% CI: 1.01–2.10) (*Table* 3). The *y*‐intercepts for having 1+ falls vs. 0 falls were between ‐2.26 and ‐2.28 and the *y*‐intercepts for having 2+ falls vs. ≤1 fall were between ‐3.86 and ‐3.89. Therefore, the odds of having 1+ injurious falls (*e*^−2.26^ = 0.104) compared with the odds of having 2+ falls (*e*^−3.86^ = 0.021) was approximately five times greater (0.10/0.20 = 4.97). The AUC values ranged from 0.50–0.54 for having 1+ injurious falls to 0.50–0.59 for having 2 + falls (*Table* S3).

Using the partial proportional odds compared with the weight proportional odds model resulted in modest changes in the odds ratios (≤0.17), although low ALM adjusted using the regression technique, a gait speed of <1.0 m/s, and the combination of low ALM adjusted for BMI with grip strength (<30 kg) became statistically significant in men, and low lean mass adjusted for height^2^ with gait speed (<1.0 m/s) became statistically significant in women (Tables S4 and S5).

### Sarcopenia—secondary analyses

In men, definitions including the 10th or 40th percentile cut offs for lean mass typically changed the odds of having an injurious fall by <0.30. Although the magnitude of the estimates were similar, using the 10th percentile cut offs resulted in a loss of statistical significance for almost all definitions, and some definitions for the 40th percentile cut offs gained significance, reflecting the impact of the prevalence of sarcopenia on the width of the CI (*Table* S6). In women, using the 40th percentile cut offs instead of the 20th percentile cut offs resulted in low lean mass adjusted for height^2^ with a gait speed of <0.8 m/s to become statistically significant with an odds ratio of 1.46 (95% CI: 1.08–1.97) (*Table* S7). Using a gait speed cut off of <1.0 m/s did not meaningfully change the results compared to the cut off of <0.8 m/s. Sarcopenia operationalized using the combination of lean mass and the chair rise test was not associated with injurious falls in men or women. Defining sarcopenia using the alternative cut offs for lean mass and gait speed or using the chair rise test did not alter the minimum and maximum AUC values observed (*Table* S3).

## Discussion

This study explored the impact of different combinations of muscle variables and different cut points on the association of sarcopenia with injurious falls. The odds of having a higher level of injurious falls were approximately two times greater in men when sarcopenia was defined as low ALM adjusted for weight, grip strength regardless of the cut off, and the combinations of low ALM adjusted for weight, BMI and using the regression technique with grip strength. In women, only sarcopenia identified as low ALM adjusted using the regression technique combined with low gait speed (<0.80 m/s) was associated with a higher level of injurious falls. Regardless of the strength of the association between sarcopenia and injurious falls, the clinical utility was limited with AUC values of <0.60 for all definitions.

The more consistent association of sarcopenia with falls in men than in women has previously been observed by two other studies with sex‐stratified analyses.^22^, ^32^ The reason for this apparent difference is unclear, but may be related to sex differences in older adults for risk factors for falls. In comparison with women, men have greater lean mass and strength^33^ and therefore may more heavily rely on their physical abilities to prevent falls as evidenced by findings that men with physical function limitations are at a higher risk of injurious falls compared with women.^34^, ^35^ Other non‐function related risk factors such as urinary incontinence are strong risk factors for falls in women but not associated with falls in men.^35^ Our study joins the growing body of evidence showing that the risk factors of falls differ in men and women and highlights the importance of conducting analyses which takes sex into account.

In men, definitions including ALM adjusted for height^2^ and grip strength were not significantly associated with injurious falls while definitions including ALM adjusted for weight, BMI, or using the residual technique and grip strength were. Few studies have compared the different lean mass adjustment techniques in the context of falls. One study observed that sarcopenia defined as unadjusted ALM, but not height^2^ adjusted ALM, was associated with falls,^21^ and one found significant associations using height^2^ adjusted definitions but not BMI adjusted definitions.^22^ A potential explanation for the attenuated association of sarcopenia when ALM is adjusted for height^2^ is because adjusting for height^2^ identifies more individuals with normal BMI values (18.5–24.9 kg/m^2^) as having low lean mass whereas the other techniques tend to identify more obese individuals (BMI > 30 kg/m^2^) as having low lean mass (*Table* S8). Obesity is an independent risk factor for falling,^36^ which may explain why lean mass adjustment techniques identifying obese individuals are more strongly associated with falls compared with adjusting for height^2^. Additionally, in our analyses, the same grip strength cut offs were applied regardless of BMI. Given that BMI is associated with greater grip strength in men, obese sarcopenic men with low grip strength may have experienced more decline compared with normal weight men.^37^ Further investigation of how ALM adjustment techniques impact the association of lean mass with health outcomes is required.

Although many sarcopenia definitions in men were associated with a two or more times greater odds of having an injurious fall, the maximum AUC value for all definitions was <0.60. AUC values are interpreted as the probability that a person who had 1+ fall was sarcopenic vs. not sarcopenic. Therefore, sarcopenia status provided little information about the risk of injurious falls over chance alone. To understand the direction of the misclassification, the sensitivity and specificity of each definition of lean mass with grip strength or gait speed for detecting falls was assessed. For 1+ falls, the sensitivity for all definitions ranged from 0.02 to 0.35 and the specificity was between 0.72 and 0.99. Therefore, all the sarcopenia definitions classified the participants not at risk for having an injurious fall well, but had a limited ability to identify those that would fall (Tables S9 and S10). The modest AUC values may in part reflect the issues of using DXA to estimate lean mass. Although considered by many to be the reference standard for measuring muscle mass for sarcopenia, DXA does not actually measure muscle mass but rather lean mass, which includes organ tissue, water, and all other non‐bone and non‐fat soft tissues in addition to muscle mass.^38^, ^39^ Unlike DXA, the D_3_‐creatine method of directly measuring muscle mass is associated with falls, performance, and mobility limitations.^40^ Therefore, if muscle mass were directly measured rather than estimated by lean mass, sarcopenia may better identify those at risk of falling.

A strength of our study was that we examined how individual components of sarcopenia definitions impacted the association between sarcopenia and injurious falls. This allowed for important findings such as how adjusting ALM for height^2^ resulted in attenuated relationships with injurious falls compared with other adjustment techniques in men. Additionally, we were able to explore how using alternative cut offs or tests such as the chair rise test impacted the association of sarcopenia with injurious falls. This strengthens our conclusion that sarcopenia is not associated with injurious falls in women as we excluded the potential that an association would be found using alternative cut offs or the chair rise test. Including the AUC analyses also provided insight about the clinical usefulness of diagnosing sarcopenia in order to identify potential fallers.

There are several limitations to our study. The results of our study may have limited generalizability. Our sample was 97% European, and therefore, our results may not be generalizable to other ethnicity groups as lean mass, grip strength, and gait speed have been shown to differ by ethnicity.^41^, ^42^, ^43^ Our analyses were not meaningfully changed when limited to just Europeans (Tables S11 and S12). Although our sample size was larger compared with most previous studies investigating sarcopenia and falls,^6^ only a small percentage of participants experienced injurious falls, and our analyses were in some cases underpowered (Tables S9 and S10). Therefore, the results of this study should be interpreted with caution as the estimates for the odds ratios and AUC values may be unstable. In order to more definitively assess the association between sarcopenia and health, individual participant data from multiple studies should be pooled to provide the required sample size. Our study may also not be directly comparable with the literature. The majority of prior studies have used all falls rather than injurious falls as an outcome.^20^, ^21^, ^22^, ^32^ An additional limitation with the outcome of injurious falls was that participants were asked about falls in the past 12 months, which is subject to recall bias compared with studies, which require participants to contact the study when a fall occurs. We also used a JTech brand electronic dynamometer to measure grip strength whereas the cut offs for grip strength were developed in studies using hydraulic Jamar brand dynamometers and the comparability between these measures has not been established.

## Conclusions

Sarcopenia operationalized as low lean mass adjusted for weight, BMI, or using the residual adjustment technique in combination with low grip strength tended to be associated with an increased level of injurious falls in men across a range of cut offs for lean mass and grip strength. Sarcopenia was generally not associated with an increased odds of injurious falls in women. The results were consistent regardless of the cut offs used for lean mass and gait speed. Using the chair rise test as an alternative to grip strength attenuated the association of sarcopenia and injurious falls in men and did not meaningfully change the association in women. In both men and women, the AUC analyses estimates for all definitions were <0.60 suggesting that sarcopenia may have limited utility in identifying potential injurious fallers. These results highlight the need for future studies to conduct sex‐stratified analyses and to explore the individual components to sarcopenia definitions to best identify people at risk of poor health. Future studies should also consider the use of AUC analyses to better understand the clinical relevance of sarcopenia for identifying people at risk of poor health.

## Conflict of interest

The authors have no conflict of interest to report.

## Supporting information


**Table S1.** Participant characteristics – weighted data
**Table S2.** Comparison of individuals with and without missing data, non‐weighted data
**Table S3.** Area under the curve statistics
**Table S4.** Association between sarcopenia and injurious falls in men using different methods of operationalizing sarcopenia – partial proportional odds model
**Table S5.** Association between sarcopenia and injurious falls in women using different methods of operationalizing sarcopenia – partial proportional odds model
**Table S6.** Association between sarcopenia and injurious falls in men using different methods of operationalizing sarcopenia
**Table S7.** Association between sarcopenia and injurious falls in women using different methods of operationalizing sarcopenia
**Table S8.** Percentage of underweight, normal weight, overweight, and obese participants for each method of adjusting for lean mass using the 20^th^ percentile cut offs
**Table S9.** Number of men with zero, one, or two or more injurious falls stratified by sarcopenia status
**Table S10.** Number of women participants with zero, one, or two or more injurious falls stratified by sarcopenia status
**Table S11.** Association between sarcopenia and injurious falls in European men using different methods of operationalizing sarcopenia
**Table S12**. Association between sarcopenia and falls in European women using different methods of operationalizing sarcopenia
