# Supplementary material for: The impact of different diagnostic criteria on the association of sarcopenia with injurious falls in the CLSA
Source: J Cachexia Sarcopenia Muscle. 2020 Sep 17;11(6):1603–13. doi: 10.1002/jcsm.12622 (PMC7749560; doi:10.1002/jcsm.12622)
Supplement: Supplementary file 1 — Table S1. Participant characteristics – weighted data Table S2. Comparison of individuals with and without missing data, non‐weighted data Table S3. Area under the curve statistics Table S4. Association between sarcopenia and injurious falls in men using different methods of operationalizing sarcopenia – partial proportional odds model Table S5. Association between sarcopenia and injurious falls in women using different methods of operationalizing sarcopenia – partial proportional odds model Table S6. Association between sarcopenia and injurious falls in men using different methods of operationalizing sarcopenia Table S7. Association between sarcopenia and injurious falls in women using different methods of operationalizing sarcopenia Table S8. Percentage of underweight, normal weight, overweight, and obese participants for each method of adjusting for lean mass using the 20th percentile cut offs Table S9. Number of men with zero, one, or two or more injurious falls stratified by sarcopenia status Table S10. Number of women participants with zero, one, or two or more injurious falls stratified by sarcopenia status Table S11. Association between sarcopenia and injurious falls in European men using different methods of operationalizing sarcopenia Table S12. Association between sarcopenia and falls in European women using different methods of operationalizing sarcopenia [file JCSM-11-1603-s001.docx]

**Supplementary Table 1 – Participant characteristics – weighted data**

| **Characteristic** | **Men (n=5162)** | | **Women (N=4774)** | |
| --- | --- | --- | --- | --- |
|  | **Mean or N** | **SE or %** | **Mean or N** | **SE or %** |
| **Age, years** | 72.5 | 5.8 | 72.4 | 5.8 |
| **European, %** | 4951 | 95.9 | 4651 | 97.4 |
| **Height, cm** | 1.7 | 0.1 | 1.6 | 0.1 |
| **Weight, kg** | 84.5 | 14.9 | 70.8 | 15.1 |
| **BMI, kg/m2** | 27.9 | 4.5 | 27.7 | 5.8 |
| **Total body fat mass, %** | 25.4 | 8.6 | 29.6 | 9.8 |
| **Appendicular lean mass, kg** | 24.5 | 3.8 | 16.3 | 3.0 |
| **ALM/height^2^** | 8.1 | 1.1 | 6.4 | 1.1 |
| **ALM/weight** | 29.2 | 3.1 | 23.2 | 2.7 |
| **ALM/BMI** | 0.9 | 0.1 | 0.6 | 0.1 |
| **ALM residuals** | 0.1 | 2.9 | -0.1 | 2.0 |
| **Gait speed, meters per second** | 0.9 | 0.2 | 0.9 | 0.2 |
| **Grip strength, kg** | 39.8 | 9.1 | 23.9 | 5.5 |
| **Chair rise test, seconds** | 13.9 | 270.3 | 14.5 | 319.4 |
| **Number of injurious falls in previous year (%)** |  |  |  |  |
| **Zero** | 4683 | 90.5 | 4161 | 86.7 |
| **One** | 375 | 7.5 | 468 | 10.1 |
| **Two or more** | 104 | 2.0 | 145 | 3.2 |
| **Self-rated general health (%)** |  |  |  |  |
| **Fair or poor** | 404 | 7.5 | 371 | 8.0 |
| **Good, very good, or excellent** | 4758 | 92.5 | 4403 | 92.0 |
| **Presence of pain or discomfort (%)** | 1631 | 32.5 | 2023 | 43.0 |
| **Self-rated hearing (%)** |  |  |  |  |
| **Fair or poor** | 912 | 16.9 | 521 | 11.1 |
| **Good, very good, or excellent** | 4250 | 83.1 | 4252 | 88.9 |
| **Urinary incontinence (%)** | 400 | 7.3 | 690 | 14.8 |
| **Household income (%)** |  |  |  |  |
| **< $20,000** | 158 | 2.9 | 387 | 8.9 |
| **≥ $20,000 < $50,000** | 1239 | 26.9 | 1765 | 40.5 |
| **≥ $50,000 <$100,000** | 2164 | 43.6 | 1497 | 35.5 |
| **≥ $100,000 < $150,000** | 842 | 16.9 | 415 | 10.5 |
| **≥ 150,000** | 462 | 9.6 | 182 | 4.5 |
| **Smoking status (%)** |  |  |  |  |
| **Current** | 263 | 5.1 | 231 | 4.9 |
| **Never or former** | 4861 | 94.9 | 4500 | 95.1 |
| **COPD (%)** | 319 | 5.8 | 372 | 7.3 |
| **Depression (%)** | 523 | 9.7 | 848 | 17.4 |
| **Neurological conditions (%)** | 349 | 7.1 | 808 | 16.8 |
| **Arthritis (%)** | 1371 | 27.0 | 2019 | 41.4 |
| **Diabetes (%)** | 1243 | 24.1 | 848 | 17.4 |
| **[Stroke (%)](file:///C:\\Users\\mayhewaj\\AppData\\Local\\Microsoft\\Windows\\Temporary%20Internet%20Files\\Content.MSO\\F5EC1CE2.xlsx#'Table%202'!A48)** | 379 | 6.8 | 303 | 6.3 |
| **[Osteoporosis (%)](file:///C:\\Users\\mayhewaj\\AppData\\Local\\Microsoft\\Windows\\Temporary%20Internet%20Files\\Content.MSO\\F5EC1CE2.xlsx#'Table%202'!A49)** | 186 | 3.7 | 1164 | 24.7 |

**Supplementary Table 2 – Comparison of individuals with and without missing data, non-weighted data**

|  | **Completers only** | | | | **Participants missing sarcopenia, injurious falls, or covariate data** | | | | | | | |
| --- | --- | --- | --- | --- | --- | --- | --- | --- | --- | --- | --- | --- |
| **Characteristic** | **Men (n=5162)** | | **Women (N=4774)** | | **Men (n=1167)** | | | | **Women (N=1525)** | | | |
|  | **Mean or N** | **SD or %** | **Mean or N** | **SD or %** | **Mean or N** | **SD or %** | **Missing data (N)** | **Missing Data (%)** | **Mean or N** | **SD or %** | **Missing data (N)** | **Missing Data (%)** |
| **Age, years** | 72.9 | 5.55 | 72.8 | 5.64 | 74.0 | 5.89 | 0 | 0.0 | 73.9 | 5.9 | 0 | 0.0 |
| **European, %** | 4951 | 95.9 | 4651 | 97.4 | 1119 | 95.9 | 0 | 0.0 | 1473 | 96.6 | 0 | 0.0 |
| **European, %** |  |  |  |  |  |  |  |  |  |  |  |  |
| **Height, cm** | 173.9 | 6.74 | 159.9 | 6.33 | 173.5 | 7.41 | 22 | 0.3 | 159.3 | 6.3 | 28 | 0.4 |
| **Weight, kg** | 84.4 | 14.07 | 70.8 | 14.29 | 85.4 | 16.04 | 23 | 0.4 | 72.0 | 16.4 | 33 | 0.5 |
| **BMI, kg/m2** | 27.9 | 4.21 | 27.7 | 5.45 | 28.3 | 4.75 | 26 | 0.4 | 28.4 | 6.3 | 35 | 0.6 |
| **Total body fat mass, kg** | 25.3 | 8.03 | 29.5 | 9.36 | 26.2 | 8.74 | 291 | 4.6 | 30.6 | 10.5 | 367 | 5.8 |
| **Appendicular lean mass, kg** | 24.4 | 3.68 | 16.3 | 2.87 | 23.8 | 3.99 | 308 | 4.9 | 16.3 | 3.1 | 383 | 6.1 |
| **ALM/height^2^** | 8.1 | 1.02 | 6.4 | 1.01 | 8.0 | 1.13 | 308 | 4.9 | 6.4 | 1.1 | 383 | 6.1 |
| **ALM/weight** | 29.1 | 2.97 | 23.3 | 2.73 | 28.4 | 3.06 | 312 | 4.9 | 22.9 | 2.9 | 386 | 6.1 |
| **ALM/BMI** | 0.9 | 0.12 | 0.6 | 0.09 | 0.8 | 0.12 | 312 | 4.9 | 0.6 | 0.1 | 386 | 6.1 |
| **Gait speed, meters per second** | 0.94 | 0.19 | 0.91 | 0.19 | 0.89 | 0.21 | 103 | 1.6 | 0.85 | 0.21 | 108 | 1.7 |
| **Grip strength, kg** | 39.5 | 8.52 | 23.9 | 5.54 | 37.5 | 8.85 | 445 | 7.0 | 22.1 | 5.4 | 773 | 12.3 |
| **Chair rise test, seconds** | 13.9 | 3.76 | 14.5 | 4.62 | 14.1 | 7.0 | 200 | 3.2 | 14.7 | 7.4 | 262 | 4.2 |
| **Number of injurious falls in previous year (%)** |  |  |  |  |  |  | 309 | 4.9 |  |  | 298 | 4.7 |
| **Zero** | 4683 | 90.5 | 4161 | 86.7 | 746 | 88.0 |  |  | 1028 | 84.8 | 1525 | 24.2 |
| **One** | 375 | 7.5 | 468 | 10.1 | 70 | 7.8 |  |  | 136 | 10.1 |  |  |
| **Two or more** | 104 | 2.0 | 145 | 3.2 | 42 | 4.2 |  |  | 63 | 5.1 |  |  |
| **Self-rated general health (%)** |  |  |  |  |  |  | 7 | 0.1 |  |  | 7 | 0.1 |
| **Fair or poor** | 404 | 7.5 | 371 | 8.0 | 199 | 17.0 |  |  | 218 | 14.4 |  |  |
| **Good, very good, or excellent** | 4758 | 92.5 | 4403 | 92.0 | 961 | 83.0 |  |  | 1300 | 85.6 |  |  |
| **Presence of pain or discomfort (%)** | 1631 | 32.5 | 2023 | 43.0 | 383 | 44.7 | 310 | 4.9 | 715 | 58.1 | 296 | 4.7 |
| **Self-rated hearing (%)** |  |  |  |  |  |  | 0 | 0.0 |  |  | 1 | 0.0 |
| **Fair or poor** | 912 | 16.9 | 521 | 11.1 | 235 | 19.7 |  |  | 222 | 13.7 |  |  |
| **Good, very good, or excellent** | 4250 | 83.1 | 4252 | 88.9 | 932 | 80.3 |  |  | 1302 | 86.3 |  |  |
| **Urinary incontinence (%)** | 400 | 7.3 | 690 | 14.8 | 124 | 10.5 | 34 | 0.5 | 290 | 21.0 | 32 | 0.5 |
| **Household income (%)** |  |  |  |  |  |  | 94 | 1.5 |  |  | 206 | 3.3 |
| **< $20,000** | 158 | 2.9 | 387 | 8.9 | 77 | 6.5 |  |  | 187 | 13.6 |  |  |
| **≥ $20,000 < $50,000** | 1239 | 26.9 | 1765 | 40.5 | 342 | 32.1 |  |  | 566 | 41.1 |  |  |
| **≥ $50,000 <$100,000** | 2164 | 43.6 | 1497 | 35.5 | 428 | 39.7 |  |  | 436 | 33.9 |  |  |
| **≥ $100,000 < $150,000** | 842 | 16.9 | 415 | 10.5 | 146 | 13.6 |  |  | 101 | 9.0 |  |  |
| **≥ 150,000** | 462 | 9.6 | 182 | 4.5 | 80 | 8.2 |  |  | 29 | 2.4 |  |  |
| **Smoking status (%)** |  |  |  |  |  |  | 10 | 0.2 |  |  | 6 | 0.1 |
| **Current** | 262 | 5.1 | 231 | 4.9 | 76 | 6.6 |  |  | 96 | 6.3 |  |  |
| **Never or former** | 4861 | 94.9 | 4500 | 95.1 | 1081 | 93.4 |  |  | 1423 | 93.7 |  |  |
| **COPD (%)** | 319 | 5.8 | 372 | 7.3 | 114 | 9.7 | 30 | 0.5 | 168 | 10.2 | 35 | 0.6 |
| **Depression (%)** | 523 | 9.7 | 848 | 17.4 | 166 | 15.3 | 57 | 0.9 | 347 | 22.8 | 57 | 0.9 |
| **Neurological conditions (%)** | 349 | 7.1 | 808 | 16.8 | 95 | 8.6 | 27 | 0.4 | 312 | 21.1 | 31 | 0.5 |
| **Arthritis (%)** | 1371 | 27.0 | 2019 | 41.4 | 296 | 30.2 | 203 | 3.2 | 679 | 52.0 | 241 | 3.8 |
| **Diabetes (%)** | 1243 | 24.1 | 848 | 17.4 | 339 | 29.4 | 29 | 0.5 | 321 | 20.2 | 31 | 0.5 |
| [**Stroke (%)**](file:///C:\Users\mayhewaj\AppData\Local\Microsoft\Windows\Temporary%20Internet%20Files\Content.MSO\F5EC1CE2.xlsx#Sheet1!A48) | 379 | 6.8 | 303 | 6.3 | 136 | 11.5 | 36 | 0.6 | 128 | 8.5 | 42 | 0.7 |
| [**Osteoporosis (%)**](file:///C:\Users\mayhewaj\AppData\Local\Microsoft\Windows\Temporary%20Internet%20Files\Content.MSO\F5EC1CE2.xlsx#Sheet1!A49) | 186 | 3.7 | 1164 | 24.7 | 66 | 6.3 | 40 | 0.6 | 378 | 26.4 | 60 | 1.0 |

**Supplementary Table 3 – Area under the curve statistics**

| **Sarcopenia definition** | | | **Men** | | | | **Women** | | | |
| --- | --- | --- | --- | --- | --- | --- | --- | --- | --- | --- |
|  |  |  | **1+ injurious fall** | | **2+ injurious falls** | | **1+ injurious all** | | **2+ injurious falls** | |
| **Lean mass percentile** | **Combination of muscle variables** | **Method of adjusting lean mass** | **Area under the curve** | **95% Confidence interval** | **Area under the curve** | **95% Confidence interval** | **Area under the curve** | **95% Confidence interval** | **Area under the curve** | **95% Confidence interval** |
| **Grip strength <30kg men, <20kg women** | | | 0.54 | 0.51 - 0.55 | 0.57 | 0.52 - 0.61 | 0.51 | 0.50 - 0.53 | 0.53 | 0.49 - 0.57 |
| **Grip strength <26kg men, <16kg women** | | | 0.52 | 0.51 - 0.54 | 0.53 | 0.50 - 0.56 | 0.50 | 0.49 - 0.51 | 0.50 | 0.48 - 0.52 |
| **Grip strength <27kg men** | | | 0.53 | 0.51 - 0.53 | 0.53 | 0.50 - 0.56 | 0.50 | 0.49 - 0.51 | 0.50 | 0.48 - 0.52 |
| **Gait speed <0.8m/s** | | | 0.54 | 0.51 - 0.53 | 0.59 | 0.55 - 0.64 | 0.54 | 0.52 - 0.56 | 0.59 | 0.55 - 0.64 |
| **Gait speed <1.0m/s** | | | 0.52 | 0.51 - 0.53 | 0.54 | 0.49 - 0.58 | 0.52 | 0.50 - 0.54 | 0.54 | 0.50 - 0.57 |
| **Chair rise ≥15 seconds** | | | 0.53 | 0.51 - 0.53 | 0.57 | 0.52 - 0.62 | 0.52 | 0.50 - 0.54 | 0.57 | 0.53 - 0.61 |
| **10th** | **Lean mass only** | **Height^2^** | 0.51 | 0.50 - 0.53 | 0.52 | 0.49 - 0.56 | 0.51 | 0.49 - 0.52 | 0.52 | 0.49 - 0.54 |
|  |  | **Weight** | 0.52 | 0.51 - 0.53 | 0.52 | 0.48 - 0.55 | 0.50 | 0.49 - 0.51 | 0.52 | 0.49 - 0.54 |
|  |  | **BMI** | 0.53 | 0.50 - 0.53 | 0.54 | 0.50 - 0.58 | 0.50 | 0.49 - 0.51 | 0.51 | 0.48 - 0.54 |
|  |  | **Residuals** | 0.52 | 0.52 - 0.55 | 0.54 | 0.50 - 0.58 | 0.50 | 0.49 - 0.51 | 0.51 | 0.49 - 0.54 |
|  | **Lean mass and grip strength <30kg men, <20kg women** | **Height^2^** | 0.51 | 0.51 - 0.53 | 0.51 | 0.49 - 0.53 | 0.50 | 0.49 - 0.51 | 0.50 | 0.49 - 0.52 |
|  |  | **Weight** | 0.51 | 0.51 - 0.54 | 0.51 | 0.49 - 0.52 | 0.50 | 0.50 - 0.51 | 0.50 | 0.49 - 0.52 |
|  |  | **BMI** | 0.51 | 0.51 - 0.54 | 0.53 | 0.50 - 0.55 | 0.51 | 0.50 - 0.52 | 0.50 | 0.48 - 0.52 |
|  |  | **Residuals** | 0.51 | 0.51 - 0.54 | 0.52 | 0.50 - 0.54 | 0.50 | 0.49 - 0.51 | 0.51 | 0.49 - 0.53 |
|  | **Lean mass and grip strength <26kg men, <16kg women** | **Height^2^** | 0.50 | 0.50 - 0.54 | 0.51 | 0.49 - 0.53 | 0.50 | 0.50 - 0.51 | 0.50 | 0.49 - 0.51 |
|  |  | **Weight** | 0.51 | 0.51 - 0.55 | 0.50 | 0.49 - 0.52 | 0.50 | 0.50 - 0.51 | 0.51 | 0.50 - 0.51 |
|  |  | **BMI** | 0.51 | 0.51 - 0.54 | 0.52 | 0.50 - 0.54 | 0.50 | 0.50 - 0.51 | 0.50 | 0.49 - 0.51 |
|  |  | **Residuals** | 0.51 | 0.51 - 0.55 | 0.51 | 0.49 - 0.53 | 0.50 | 0.50 - 0.50 | 0.50 | 0.49 - 0.51 |
|  | **Lean mass and grip strength <27kg men** | **Height^2^** | 0.51 | 0.51 - 0.56 | 0.51 | 0.49 - 0.52 | 0.50 | 0.50 - 0.51 | 0.50 | 0.49 - 0.51 |
|  |  | **Weight** | 0.51 | 0.52 - 0.56 | 0.50 | 0.49 - 0.51 | 0.50 | 0.50 - 0.51 | 0.51 | 0.50 - 0.51 |
|  |  | **BMI** | 0.51 | 0.51 - 0.56 | 0.52 | 0.50 - 0.54 | 0.50 | 0.50 - 0.51 | 0.50 | 0.49 - 0.51 |
|  |  | **Residuals** | 0.51 | 0.52 - 0.56 | 0.51 | 0.49 - 0.52 | 0.50 | 0.50 - 0.50 | 0.50 | 0.49 - 0.51 |
|  | **Lean mass and gait speed <0.8m/s** | **Height^2^** | 0.51 | 0.5 - 0.54 | 0.52 | 0.50 - 0.54 | 0.51 | 0.50 - 0.51 | 0.53 | 0.51 - 0.55 |
|  |  | **Weight** | 0.51 | 0.52 - 0.56 | 0.51 | 0.49 - 0.53 | 0.50 | 0.49 - 0.51 | 0.52 | 0.50 - 0.55 |
|  |  | **BMI** | 0.51 | 0.52 - 0.55 | 0.52 | 0.49 - 0.54 | 0.50 | 0.49 - 0.51 | 0.52 | 0.49 - 0.54 |
|  |  | **Residuals** | 0.52 | 0.51 - 0.55 | 0.52 | 0.50 - 0.55 | 0.51 | 0.50 - 0.52 | 0.53 | 0.51 - 0.56 |
|  | **Lean mass and gait speed <1.0m/s** | **Height^2^** | 0.51 | 0.51 - 0.55 | 0.52 | 0.49 - 0.55 | 0.51 | 0.50 - 0.52 | 0.53 | 0.50 - 0.55 |
|  |  | **Weight** | 0.52 | 0.51 - 0.55 | 0.51 | 0.48 - 0.54 | 0.50 | 0.49 - 0.51 | 0.52 | 0.49 - 0.54 |
|  |  | **BMI** | 0.52 | 0.51 - 0.55 | 0.53 | 0.50 - 0.56 | 0.50 | 0.49 - 0.51 | 0.51 | 0.49 - 0.54 |
|  |  | **Residuals** | 0.52 | 0.51 - 0.55 | 0.53 | 0.49 - 0.56 | 0.50 | 0.49 - 0.51 | 0.52 | 0.49 - 0.55 |
|  | **Lean mass and chair rise ≥ 15 seconds** | **Height^2^** | 0.51 | 0.51 - 0.55 | 0.51 | 0.49 - 0.54 | 0.51 | 0.50 - 0.52 | 0.53 | 0.50 - 0.55 |
|  |  | **Weight** | 0.51 | 0.51 - 0.55 | 0.51 | 0.49 - 0.54 | 0.51 | 0.50 - 0.52 | 0.53 | 0.50 - 0.55 |
|  |  | **BMI** | 0.52 | 0.51 - 0.55 | 0.54 | 0.51 - 0.57 | 0.50 | 0.49 - 0.51 | 0.52 | 0.49 - 0.54 |
|  |  | **Residuals** | 0.51 | 0.51 - 0.55 | 0.52 | 0.49 - 0.55 | 0.50 | 0.49 - 0.51 | 0.53 | 0.50 - 0.55 |
| **20th** | **Lean mass only** | **Height^2^** | 0.52 | 0.50 - 0.54 | 0.52 | 0.48 - 0.56 | 0.50 | 0.49 - 0.52 | 0.52 | 0.49 - 0.56 |
|  |  | **Weight** | 0.54 | 0.51 - 0.55 | 0.53 | 0.49 - 0.58 | 0.51 | 0.49 - 0.52 | 0.55 | 0.51 - 0.59 |
|  |  | **BMI** | 0.52 | 0.50 - 0.53 | 0.54 | 0.50 - 0.59 | 0.51 | 0.49 - 0.53 | 0.56 | 0.52 - 0.60 |
|  |  | **Residuals** | 0.53 | 0.51 - 0.54 | 0.52 | 0.48 - 0.56 | 0.51 | 0.49 - 0.53 | 0.53 | 0.49 - 0.57 |
|  | **Lean mass and grip strength <30kg men, <20kg women** | **Height^2^** | 0.51 | 0.51 - 0.55 | 0.52 | 0.49 - 0.54 | 0.51 | 0.49 - 0.52 | 0.51 | 0.49 - 0.54 |
|  |  | **Weight** | 0.52 | 0.51 - 0.55 | 0.52 | 0.49 - 0.55 | 0.51 | 0.50 - 0.51 | 0.51 | 0.49 - 0.53 |
|  |  | **BMI** | 0.52 | 0.51 - 0.55 | 0.54 | 0.51 - 0.57 | 0.50 | 0.49 - 0.51 | 0.52 | 0.49 - 0.55 |
|  |  | **Residuals** | 0.52 | 0.51 - 0.55 | 0.52 | 0.49 - 0.54 | 0.51 | 0.50 - 0.52 | 0.52 | 0.50 - 0.54 |
|  | **Lean mass and grip strength <26kg men, <16kg women** | **Height^2^** | 0.50 | 0.51 - 0.55 | 0.51 | 0.49 - 0.53 | 0.50 | 0.49 - 0.51 | 0.50 | 0.49 - 0.51 |
|  |  | **Weight** | 0.52 | 0.51 - 0.55 | 0.52 | 0.50 - 0.55 | 0.50 | 0.50 - 0.51 | 0.51 | 0.50 - 0.51 |
|  |  | **BMI** | 0.52 | 0.51 - 0.55 | 0.53 | 0.50 - 0.55 | 0.50 | 0.49 - 0.51 | 0.50 | 0.49 - 0.51 |
|  |  | **Residuals** | 0.51 | 0.51 - 0.55 | 0.51 | 0.49 - 0.53 | 0.50 | 0.50 - 0.51 | 0.50 | 0.49 - 0.51 |
|  | **Lean mass and grip strength <27kg men** | **Height^2^** | 0.51 | 0.51 - 0.55 | 0.51 | 0.49 - 0.53 | 0.50 | 0.49 - 0.51 | 0.50 | 0.49 - 0.51 |
|  |  | **Weight** | 0.52 | 0.51 - 0.55 | 0.52 | 0.50 - 0.55 | 0.50 | 0.50 - 0.51 | 0.51 | 0.50 - 0.51 |
|  |  | **BMI** | 0.52 | 0.51 - 0.55 | 0.52 | 0.50 - 0.55 | 0.50 | 0.49 - 0.51 | 0.50 | 0.49 - 0.51 |
|  |  | **Residuals** | 0.52 | 0.51 - 0.55 | 0.51 | 0.49 - 0.53 | 0.50 | 0.50 - 0.51 | 0.50 | 0.49 - 0.51 |
|  | **Lean mass and gait speed <0.8m/s** | **Height^2^** | 0.51 | 0.51 - 0.55 | 0.52 | 0.49 - 0.55 | 0.51 | 0.50 - 0.52 | 0.54 | 0.51 - 0.57 |
|  |  | **Weight** | 0.52 | 0.51 - 0.55 | 0.53 | 0.50 - 0.56 | 0.51 | 0.49 - 0.52 | 0.55 | 0.52 - 0.58 |
|  |  | **BMI** | 0.51 | 0.51 - 0.55 | 0.54 | 0.50 - 0.57 | 0.51 | 0.50 - 0.52 | 0.55 | 0.52 - 0.59 |
|  |  | **Residuals** | 0.52 | 0.51 - 0.55 | 0.53 | 0.50 - 0.56 | 0.52 | 0.50 - 0.53 | 0.55 | 0.52 - 0.58 |
|  | **Lean mass and gait speed <1.0m/s** | **Height^2^** | 0.51 | 0.51 - 0.55 | 0.5 | 0.47 - 0.54 | 0.51 | 0.49 - 0.52 | 0.54 | 0.50 - 0.57 |
|  |  | **Weight** | 0.54 | 0.51 - 0.55 | 0.53 | 0.49 - 0.57 | 0.51 | 0.49 - 0.52 | 0.54 | 0.51 - 0.58 |
|  |  | **BMI** | 0.52 | 0.51 - 0.55 | 0.55 | 0.50 - 0.59 | 0.51 | 0.49 - 0.53 | 0.55 | 0.52 - 0.59 |
|  |  | **Residuals** | 0.53 | 0.51 - 0.55 | 0.51 | 0.48 - 0.55 | 0.51 | 0.50 - 0.53 | 0.53 | 0.50 - 0.57 |
|  | **Lean mass and chair rise ≥ 15 seconds** | **Height^2^** | 0.51 | 0.51 - 0.55 | 0.52 | 0.49 - 0.55 | 0.51 | 0.50 - 0.52 | 0.55 | 0.51 - 0.58 |
|  |  | **Weight** | 0.53 | 0.51 - 0.55 | 0.52 | 0.49 - 0.56 | 0.51 | 0.49 - 0.52 | 0.55 | 0.52 - 0.58 |
|  |  | **BMI** | 0.52 | 0.51 - 0.55 | 0.55 | 0.51 - 0.59 | 0.52 | 0.50 - 0.53 | 0.56 | 0.52 - 0.59 |
|  |  | **Residuals** | 0.52 | 0.51 - 0.55 | 0.51 | 0.48 - 0.54 | 0.51 | 0.50 - 0.53 | 0.55 | 0.52 - 0.58 |
| **40th** | **Lean mass only** | **Height^2^** | 0.53 | 0.51 - 0.54 | 0.53 | 0.48 - 0.58 | 0.50 | 0.48 - 0.52 | 0.51 | 0.47 - 0.55 |
|  |  | **Weight** | 0.54 | 0.5 - 0.53 | 0.55 | 0.50 - 0.59 | 0.52 | 0.50 - 0.54 | 0.52 | 0.48 - 0.56 |
|  |  | **BMI** | 0.54 | 0.52 - 0.55 | 0.56 | 0.51 - 0.61 | 0.52 | 0.50 - 0.54 | 0.55 | 0.51 - 0.59 |
|  |  | **Residuals** | 0.54 | 0.51 - 0.54 | 0.54 | 0.50 - 0.59 | 0.51 | 0.49 - 0.53 | 0.52 | 0.48 - 0.56 |
|  | **Lean mass and grip strength <30kg men, <20kg women** | **Height^2^** | 0.53 | 0.51 - 0.55 | 0.56 | 0.52 - 0.60 | 0.51 | 0.50 - 0.52 | 0.52 | 0.49 - 0.55 |
|  |  | **Weight** | 0.53 | 0.51 - 0.55 | 0.55 | 0.51 - 0.58 | 0.51 | 0.49 - 0.52 | 0.52 | 0.49 - 0.55 |
|  |  | **BMI** | 0.53 | 0.51 - 0.55 | 0.55 | 0.52 - 0.59 | 0.50 | 0.49 - 0.52 | 0.52 | 0.49 - 0.55 |
|  |  | **Residuals** | 0.53 | 0.51 - 0.55 | 0.54 | 0.51 - 0.58 | 0.51 | 0.49 - 0.52 | 0.52 | 0.49 - 0.55 |
|  | **Lean mass and grip strength <26kg men, <16kg women** | **Height^2^** | 0.52 | 0.51 - 0.55 | 0.52 | 0.50 - 0.55 | 0.51 | 0.50 - 0.52 | 0.51 | 0.49 - 0.52 |
|  |  | **Weight** | 0.52 | 0.51 - 0.55 | 0.53 | 0.51 - 0.56 | 0.50 | 0.49 - 0.51 | 0.50 | 0.49 - 0.52 |
|  |  | **BMI** | 0.52 | 0.51 - 0.55 | 0.53 | 0.50 - 0.56 | 0.50 | 0.49 - 0.51 | 0.51 | 0.49 - 0.52 |
|  |  | **Residuals** | 0.52 | 0.51 - 0.55 | 0.53 | 0.50 - 0.56 | 0.50 | 0.49 - 0.51 | 0.50 | 0.49 - 0.52 |
|  | **Lean mass and grip strength <27kg men** | **Height^2^** | 0.52 | 0.51 - 0.55 | 0.52 | 0.50 - 0.55 | 0.51 | 0.50 - 0.52 | 0.51 | 0.49 - 0.52 |
|  |  | **Weight** | 0.52 | 0.51 - 0.55 | 0.53 | 0.50 - 0.56 | 0.50 | 0.49 - 0.51 | 0.50 | 0.49 - 0.52 |
|  |  | **BMI** | 0.53 | 0.51 - 0.55 | 0.53 | 0.50 - 0.56 | 0.50 | 0.49 - 0.51 | 0.51 | 0.49 - 0.52 |
|  |  | **Residuals** | 0.52 | 0.51 - 0.55 | 0.53 | 0.50 - 0.56 | 0.50 | 0.49 - 0.51 | 0.50 | 0.49 - 0.52 |
|  | **Lean mass and gait speed <0.8m/s** | **Height^2^** | 0.52 | 0.51 - 0.55 | 0.53 | 0.50 - 0.57 | 0.53 | 0.51 - 0.54 | 0.56 | 0.52 - 0.59 |
|  |  | **Weight** | 0.53 | 0.51 - 0.55 | 0.57 | 0.53 - 0.62 | 0.52 | 0.50 - 0.53 | 0.56 | 0.52 - 0.59 |
|  |  | **BMI** | 0.52 | 0.51 - 0.55 | 0.57 | 0.53 - 0.62 | 0.52 | 0.51 - 0.54 | 0.58 | 0.54 - 0.61 |
|  |  | **Residuals** | 0.53 | 0.51 - 0.55 | 0.56 | 0.52 - 0.59 | 0.52 | 0.51 - 0.54 | 0.57 | 0.53 - 0.60 |
|  | **Lean mass and gait speed <1.0m/s** | **Height^2^** | 0.53 | 0.51 - 0.55 | 0.54 | 0.49 - 0.58 | 0.52 | 0.50 - 0.54 | 0.53 | 0.49 - 0.57 |
|  |  | **Weight** | 0.54 | 0.51 - 0.55 | 0.55 | 0.50 - 0.60 | 0.52 | 0.50 - 0.54 | 0.53 | 0.49 - 0.57 |
|  |  | **BMI** | 0.53 | 0.51 - 0.55 | 0.57 | 0.52 - 0.62 | 0.52 | 0.50 - 0.54 | 0.56 | 0.51 - 0.60 |
|  |  | **Residuals** | 0.54 | 0.51 - 0.55 | 0.56 | 0.51 - 0.60 | 0.52 | 0.50 - 0.54 | 0.54 | 0.50 - 0.58 |
|  | **Lean mass and chair rise ≥ 15 seconds** | **Height^2^** | 0.52 | 0.51 - 0.55 | 0.55 | 0.51 - 0.59 | 0.51 | 0.50 - 0.53 | 0.54 | 0.50 - 0.57 |
|  |  | **Weight** | 0.54 | 0.51 - 0.55 | 0.56 | 0.52 - 0.61 | 0.52 | 0.50 - 0.54 | 0.56 | 0.52 - 0.59 |
|  |  | **BMI** | 0.53 | 0.51 - 0.55 | 0.59 | 0.54 - 0.64 | 0.51 | 0.50 - 0.53 | 0.57 | 0.53 - 0.61 |
|  |  | **Residuals** | 0.53 | 0.51 - 0.55 | 0.57 | 0.52 - 0.61 | 0.52 | 0.50 - 0.53 | 0.55 | 0.52 - 0.59 |

**Supplementary Table 4 – Association between sarcopenia and injurious falls in men using different methods of operationalizing sarcopenia – partial proportional odds model**

| **Sarcopenia definition** | | **Number of participants with sarcopenia (%)** | **Y-intercept for 1+ injurious falls versus no falls** | **Y-intercept for 2+ injurious falls versus zero or one falls** | **Odds of falling** | **95% Confidence interval** | **p-value**  **Combination of muscle variables** |
| --- | --- | --- | --- | --- | --- | --- | --- |
|  |  |  |  |  |  |  |  |
| **Combination of muscle variables** | **Method of adjusting lean mass** |  |  |  |  |  |  |
| **Lean mass only (20th percentile)** | **Height**^2^ | 1016 (19.7) | -2.71 | -4.62 | 1.18 | 0.93 - 1.48 | 0.167 |
|  | **Weight** | 995 (19.3) | -2.72 | -4.63 | 1.38 | 1.10 - 1.72 | 0.005* |
|  | **BMI** | 994 (19.3) | -2.69 | -4.60 | 1.09 | 0.86 - 1.37 | 0.471 |
|  | **Residuals** | 1015 (19.7) | -2.72 | -4.64 | 1.31 | 1.05 - 1.64 | 0.017* |
| **Grip strength only <20kg** | | 668 (12.9) | -2.71 | -4.62 | 1.49 | 1.16 - 1.91 | 0.002* |
| **Grip strength only <16kg** | | 257 (5.0) | -2.69 | -4.60 | 1.72 | 1.22 - 2.44 | 0.002* |
| **Gait speed only <0.8m/s** | | 1138 (22.0) | -2.70 | -4.62 | 1.25 | 1 - 1.57 | 0.046* |
| **Lean mass (20th percentile) and grip strength <20kg** | **Height**^2^ | 244 (4.7) | -2.68 | -4.59 | 1.19 | 0.8 - 1.77 | 0.391 |
|  | **Weight** | 232 (4.5) | -2.68 | -4.59 | 1.50 | 1.04 - 2.18 | 0.031* |
|  | **BMI** | 294 (5.7) | -2.69 | -4.60 | 1.46 | 1.04 - 2.06 | 0.029* |
|  | **Residuals** | 215 (4.2) | -2.68 | -4.60 | 1.61 | 1.1 - 2.36 | 0.015* |
| **Lean mass (20th percentile) and grip strength <16kg** | **Height**^2^ | 106 (2.1) | -2.68 | -4.59 | 1.14 | 0.64 - 2.05 | 0.649 |
|  | **Weight** | 105 (2) | -2.68 | -4.59 | 2.00 | 1.23 - 3.26 | 0.005* |
|  | **BMI** | 123 (2.4) | -2.68 | -4.59 | 1.91 | 1.2 - 3.05 | 0.006* |
|  | **Residuals** | 90 (1.7) | -2.68 | -4.59 | 1.90 | 1.12 - 3.22 | 0.017* |
| **Lean mass (20th percentile) and gait speed <0.8m/s** | **Height**^2^ | 254 (4.9) | -2.68 | -4.59 | 1.12 | 0.75 - 1.66 | 0.580 |
|  | **Weight** | 348 (6.7) | -2.68 | -4.59 | 1.22 | 0.87 - 1.7 | 0.248 |
|  | **BMI** | 363 (7) | -2.68 | -4.59 | 0.98 | 0.69 - 1.39 | 0.929 |
|  | **Residuals** | 280 (5.4) | -2.68 | -4.60 | 1.35 | 0.95 - 1.93 | 0.097 |

* Statistically significant (p<0.05)

**Supplementary Table 5 – Association between sarcopenia and injurious falls in women using different methods of operationalizing sarcopenia – partial proportional odds model**

| **Sarcopenia definition** | | **Number of participants with sarcopenia (%)** | **Y-intercept for 1+ injurious falls versus no falls** | **Y-intercept for 2+ injurious falls versus zero or one falls** | **Odds of falling** | **95% Confidence interval** | **p-value** |
| --- | --- | --- | --- | --- | --- | --- | --- |
|  |  |  |  |  |  |  |  |
| **Combination of muscle variables** | **Method of adjusting lean mass** |  |  |  |  |  |  |
| **Lean mass only (20th percentile)** | **Height**^2^ | 960 (20.1) | -2.32 | -4.01 | 0.92 | 0.74 - 1.15 | 0.460 |
|  | **Weight** | 936 (19.6) | -2.28 | -3.97 | 1.04 | 0.87 - 1.23 | 0.460 |
|  | **BMI** | 930 (19.5) | -2.29 | -3.98 | 1.02 | 0.83 - 1.26 | 0.843 |
|  | **Residuals** | 950 (19.9) | -2.30 | -4.00 | 1.09 | 0.88 - 1.35 | 0.419 |
| **Grip strength only <20kg** | | 1092 (22.9) | -2.29 | -3.98 | 1.03 | 0.84 - 1.26 | 0.792 |
| **Grip strength only <16kg** | | 330 (6.9) | -2.29 | -3.98 | 0.91 | 0.65 - 1.27 | 0.562 |
| **Gait speed only <0.8m/s** | | 1394 (29.2) | -2.31 | -4.00 | 1.17 | 0.96 - 1.42 | 0.120 |
| **Lean mass (20th percentile) and grip strength <20kg** | **Height**^2^ | 329 (6.9) | -2.29 | -3.99 | 1.15 | 0.83 - 1.60 | 0.397 |
|  | **Weight** | 300 (6.3) | -2.28 | -3.98 | 0.65 | 0.44 - 0.95 | 0.026 |
|  | **BMI** | 361 (7.6) | -2.29 | -3.98 | 0.85 | 0.61 - 1.18 | 0.328 |
|  | **Residuals** | 280 (5.9) | -2.29 | -3.99 | 1.16 | 0.82 - 1.6 | 0.404 |
| **Lean mass (20th percentile) and grip strength <16kg** | **Height**^2^ | 118 (2.5) | -2.29 | -3.98 | 0.96 | 0.56 - 1.64 | 0.867 |
|  | **Weight** | 85 (1.8) | -2.29 | -3.98 | 0.43 | 0.20 - 0.95 | 0.038 |
|  | **BMI** | 121 (2.5) | -2.29 | -3.98 | 0.74 | 0.42 - 1.29 | 0.283 |
|  | **Residuals** | 86 (1.8) | -2.29 | -3.98 | 0.73 | 0.37 - 1.44 | 0.368 |
| **Lean mass (20th percentile) and gait speed <0.8m/s** | **Height**^2^ | 265 (5.6) | -2.30 | -3.99 | 1.42 | 1.01 - 1.99 | 0.041 |
|  | **Weight** | 409 (8.6) | -2.29 | -3.98 | 0.91 | 0.78 - 1.24 | 0.556 |
|  | **BMI** | 393 (8.2) | -2.29 | -3.98 | 1.03 | 0.77 - 1.39 | 0.834 |
|  | **Residuals** | 304 (6.4) | -2.29 | -3.99 | 1.43 | 1.05 - 1.95 | 0.024 |

* Statistically significant (p<0.05)

**Supplementary Table 6 - Association between sarcopenia and injurious falls in men using different methods of operationalizing sarcopenia**

| **Sarcopenia definition** | | | **Number of participants with sarcopenia (%)** | **Intercept 1** | **Intercept 2** | **Odds of injurious fall** | **95% Confidence interval** | **p-value** |
| --- | --- | --- | --- | --- | --- | --- | --- | --- |
| **Lean mass percentile** | **Combination of muscle variables** | **Method of adjusting lean mass** |  |  |  |  |  |  |
| **Not applicable** | **Grip strength <30kg** | | 668 (12.9) | -2.70 | -4.32 | 1.43 | 1.02 - 1.99 | 0.035 |
|  | **Grip strength <26kg** | | 257 (5.0) | -2.69 | -4.31 | 1.78 | 1.12 - 2.82 | 0.014 |
|  | **Grip strength <27kg** | | 333 (6.5) | -2.69 | -4.32 | 1.77 | 1.18 - 2.67 | 0.006 |
|  | **Gait speed <0.8m/s** | | 1138 (22.0) | -2.71 | -4.33 | 1.30 | 0.98 - 1.73 | 0.073 |
|  | **Gait speed <1.0m/s** | | 3350 (64.9) | -2.64 | -4.26 | 0.93 | 0.71 - 1.21 | 0.593 |
|  | **Chair rise ≥15 seconds** | | 1859 (36.0) | -2.69 | -4.30 | 1.02 | 0.79 - 1.31 | 0.898 |
| **10th** | **Lean mass only** | **Height^2^** | 500 (9.7) | -2.69 | -4.31 | 1.17 | 0.79 - 1.74 | 0.423 |
|  |  | **Weight** | 493 (9.6) | -2.69 | -4.31 | 1.23 | 0.85 - 1.80 | 0.275 |
|  |  | **BMI** | 481 (9.3) | -2.70 | -4.32 | 1.40 | 0.97 - 2.02 | 0.072 |
|  |  | **Residuals** | 507 (9.8) | -2.70 | -4.32 | 1.39 | 0.96 - 2.02 | 0.079 |
|  | **Lean mass and grip strength <30kg** | **Height^2^** | 135 (2.6) | -2.69 | -4.30 | 1.52 | 0.79 - 2.92 | 0.207 |
|  |  | **Weight** | 142 (2.8) | -2.68 | -4.30 | 1.51 | 0.83 - 2.76 | 0.180 |
|  |  | **BMI** | 178 (3.4) | -2.68 | -4.30 | 1.48 | 0.85 - 2.58 | 0.170 |
|  |  | **Residuals** | 119 (2.3) | -2.68 | -4.30 | 1.39 | 0.70 - 2.78 | 0.346 |
|  | **Lean mass and grip strength <26kg** | **Height^2^** | 60 (1.2) | -2.68 | -4.30 | 1.48 | 0.56 - 3.92 | 0.427 |
|  |  | **Weight** | 72 (1.4) | -2.68 | -4.30 | 1.68 | 0.76 - 3.72 | 0.197 |
|  |  | **BMI** | 82 (1.6) | -2.68 | -4.30 | 1.77 | 0.83 - 3.78 | 0.138 |
|  |  | **Residuals** | 57 (1.1) | -2.68 | -4.30 | 1.41 | 0.54 - 3.68 | 0.488 |
|  | **Lean mass and grip strength <27kg** | **Height^2^** | 75 (1.5) | -2.68 | -4.30 | 1.99 | 0.91 - 4.38 | 0.085 |
|  |  | **Weight** | 85 (1.6) | -2.68 | -4.30 | 1.95 | 0.98 - 3.90 | 0.058 |
|  |  | **BMI** | 98 (1.9) | -2.68 | -4.31 | 2.01 | 1.04 - 3.91 | 0.038 |
|  |  | **Residuals** | 71 (1.4) | -2.68 | -4.30 | 1.55 | 0.67 - 3.59 | 0.303 |
|  | **Lean mass and gait speed <0.8m/s** | **Height^2^** | 148 (2.9) | -2.68 | -4.30 | 1.13 | 0.58 - 2.18 | 0.725 |
|  |  | **Weight** | 200 (3.9) | -2.68 | -4.30 | 1.17 | 0.67 - 2.04 | 0.579 |
|  |  | **BMI** | 205 (4.0) | -2.68 | -4.30 | 1.21 | 0.71 - 2.08 | 0.484 |
|  |  | **Residuals** | 158 (3.1) | -2.68 | -4.30 | 1.48 | 0.82 - 2.67 | 0.194 |
|  | **Lean mass and gait speed <1.0m/s** | **Height^2^** | 360 (7.0) | -2.69 | -4.31 | 1.15 | 0.73 - 1.81 | 0.540 |
|  |  | **Weight** | 402 (7.8) | -2.69 | -4.30 | 1.18 | 0.78 - 1.79 | 0.429 |
|  |  | **BMI** | 395 (7.7) | -2.69 | -4.31 | 1.26 | 0.84 - 1.89 | 0.271 |
|  |  | **Residuals** | 372 (7.2) | -2.69 | -4.31 | 1.29 | 0.85 - 1.98 | 0.235 |
|  | **Lean mass and chair rise ≥15 seconds** | **Height^2^** | 227 (4.4) | -2.69 | -4.30 | 1.16 | 0.67 - 2.01 | 0.590 |
|  |  | **Weight** | 252 (4.9) | -2.68 | -4.30 | 1.29 | 0.79 - 2.12 | 0.305 |
|  |  | **BMI** | 231 (4.5) | -2.69 | -4.31 | 1.58 | 0.97 - 2.57 | 0.065 |
|  |  | **Residuals** | 251 (4.9) | -2.69 | -4.30 | 1.22 | 0.73 - 2.05 | 0.444 |
| **20th** | **Lean mass only** | **Height^2^** | 1016 (19.7) | -2.71 | -4.33 | 1.18 | 0.88 - 1.59 | 0.276 |
|  |  | **Weight** | 995 (19.3) | -2.72 | -4.34 | 1.43 | 1.07 - 1.90 | 0.016 |
|  |  | **BMI** | 994 (19.3) | -2.69 | -4.31 | 1.06 | 0.78 - 1.44 | 0.703 |
|  |  | **Residuals** | 1015 (19.7) | -2.72 | -4.34 | 1.30 | 0.97 - 1.73 | 0.080 |
|  | **Lean mass and grip strength <30kg** | **Height^2^** | 244 (4.7) | -2.68 | -4.30 | 1.15 | 0.67 - 1.97 | 0.615 |
|  |  | **Weight** | 232 (4.5) | -2.68 | -4.30 | 1.64 | 1.01 - 2.66 | 0.048 |
|  |  | **BMI** | 294 (5.7) | -2.69 | -4.31 | 1.51 | 0.96 - 2.36 | 0.073 |
|  |  | **Residuals** | 215 (4.2) | -2.69 | -4.30 | 1.66 | 1.00 - 2.75 | 0.049 |
|  | **Lean mass and grip strength <26kg** | **Height^2^** | 106 (2.1) | -2.68 | -4.30 | 1.18 | 0.54 - 2.60 | 0.677 |
|  |  | **Weight** | 105 (2.0) | -2.68 | -4.30 | 2.14 | 1.12 - 4.09 | 0.021 |
|  |  | **BMI** | 123 (2.4) | -2.69 | -4.31 | 1.97 | 1.06 - 3.63 | 0.031 |
|  |  | **Residuals** | 90 (1.7) | -2.68 | -4.30 | 2.07 | 1.03 - 4.15 | 0.040 |
|  | **Lean mass and grip strength <27kg** | **Height^2^** | 132 (2.6) | -2.68 | -4.30 | 1.45 | 0.75 - 2.82 | 0.271 |
|  |  | **Weight** | 129 (2.5) | -2.68 | -4.31 | 2.32 | 1.31 - 4.10 | 0.004 |
|  |  | **BMI** | 152 (2.9) | -2.69 | -4.31 | 2.11 | 1.23 - 3.62 | 0.007 |
|  |  | **Residuals** | 115 (2.2) | -2.68 | -4.30 | 2.14 | 1.16 - 3.96 | 0.015 |
|  | **Lean mass and gait speed <0.8m/s** | **Height^2^** | 254 (4.9) | -2.68 | -4.30 | 1.10 | 0.65 - 1.86 | 0.732 |
|  |  | **Weight** | 348 (6.7) | -2.68 | -4.30 | 1.27 | 0.82 - 1.96 | 0.283 |
|  |  | **BMI** | 363 (7.0) | -2.68 | -4.30 | 1.01 | 0.65 - 1.59 | 0.961 |
|  |  | **Residuals** | 280 (5.4) | -2.68 | -4.30 | 1.30 | 0.81 - 2.10 | 0.276 |
|  | **Lean mass and gait speed <1.0m/s** | **Height^2^** | 704 (13.6) | -2.69 | -4.31 | 1.10 | 0.78 - 1.56 | 0.576 |
|  |  | **Weight** | 759 (14.7) | -2.70 | -4.32 | 1.26 | 0.91 - 1.74 | 0.158 |
|  |  | **BMI** | 785 (15.2) | -2.68 | -4.30 | 1.04 | 0.75 - 1.44 | 0.828 |
|  |  | **Residuals** | 730 (14.1) | -2.70 | -4.32 | 1.22 | 0.87 - 1.69 | 0.246 |
|  | **Lean mass and chair rise ≥15 seconds** | **Height^2^** | 431 (8.3) | -2.69 | -4.31 | 1.12 | 0.74 - 1.71 | 0.582 |
|  |  | **Weight** | 466 (9.0) | -2.69 | -4.31 | 1.37 | 0.94 – 2.00 | 0.105 |
|  |  | **BMI** | 421 (8.2) | -2.69 | -4.31 | 1.30 | 0.87 - 1.93 | 0.200 |
|  |  | **Residuals** | 466 (9.0) | -2.69 | -4.31 | 1.21 | 0.81 - 1.79 | 0.354 |
| **40th** | **Lean mass only** | **Height^2^** | 2044 (39.6) | -2.72 | -4.34 | 1.12 | 0.87 - 1.44 | 0.395 |
|  |  | **Weight** | 2023 (39.2) | -2.74 | -4.36 | 1.23 | 0.96 - 1.59 | 0.104 |
|  |  | **BMI** | 2007 (38.9) | -2.72 | -4.34 | 1.14 | 0.89 - 1.47 | 0.303 |
|  |  | **Residuals** | 2037 (39.5) | -2.74 | -4.36 | 1.20 | 0.93 - 1.53 | 0.162 |
|  | **Lean mass and grip strength <30kg** | **Height^2^** | 394 (7.6) | -2.69 | -4.31 | 1.58 | 1.05 - 2.36 | 0.027 |
|  |  | **Weight** | 389 (7.5) | -2.69 | -4.31 | 1.48 | 0.98 - 2.22 | 0.059 |
|  |  | **BMI** | 439 (8.5) | -2.69 | -4.31 | 1.55 | 1.06 - 2.28 | 0.025 |
|  |  | **Residuals** | 388 (7.5) | -2.69 | -4.31 | 1.57 | 1.05 - 2.35 | 0.028 |
|  | **Lean mass and grip strength <26kg** | **Height^2^** | 165 (3.2) | -2.69 | -4.30 | 1.71 | 0.97 - 3.01 | 0.063 |
|  |  | **Weight** | 157 (3.0) | -2.68 | -4.30 | 1.85 | 1.05 - 3.24 | 0.032 |
|  |  | **BMI** | 175 (3.4) | -2.68 | -4.31 | 2.03 | 1.20 - 3.45 | 0.008 |
|  |  | **Residuals** | 162 (3.1) | -2.68 | -4.30 | 1.93 | 1.11 - 3.34 | 0.020 |
|  | **Lean mass and grip strength <27kg** | **Height^2^** | 209 (4.0) | -2.69 | -4.31 | 1.80 | 1.09 - 2.98 | 0.022 |
|  |  | **Weight** | 205 (4.0) | -2.68 | -4.31 | 1.95 | 1.20 - 3.18 | 0.007 |
|  |  | **BMI** | 228 (4.4) | -2.69 | -4.31 | 2.10 | 1.32 - 3.34 | 0.002 |
|  |  | **Residuals** | 208 (4.0) | -2.69 | -4.31 | 2.00 | 1.23 - 3.27 | 0.005 |
|  | **Lean mass and gait speed <0.8m/s** | **Height^2^** | 491 (9.5) | -2.69 | -4.31 | 1.17 | 0.79 - 1.73 | 0.441 |
|  |  | **Weight** | 600 (11.6) | -2.69 | -4.31 | 1.25 | 0.88 - 1.79 | 0.218 |
|  |  | **BMI** | 601 (11.6) | -2.69 | -4.30 | 1.16 | 0.81 - 1.66 | 0.428 |
|  |  | **Residuals** | 536 (10.4) | -2.69 | -4.31 | 1.28 | 0.89 - 1.85 | 0.189 |
|  | **Lean mass and gait speed <1.0m/s** | **Height^2^** | 1367 (26.5) | -2.72 | -4.33 | 1.19 | 0.91 - 1.57 | 0.211 |
|  |  | **Weight** | 1467 (28.4) | -2.70 | -4.32 | 1.13 | 0.86 - 1.49 | 0.372 |
|  |  | **BMI** | 1484 (28.7) | -2.69 | -4.31 | 1.08 | 0.82 - 1.42 | 0.578 |
|  |  | **Residuals** | 1412 (27.4) | -2.72 | -4.34 | 1.24 | 0.95 - 1.63 | 0.113 |
|  | **Lean mass and chair rise ≥15 seconds** | **Height^2^** | 796 (15.4) | -2.69 | -4.31 | 1.09 | 0.78 - 1.52 | 0.608 |
|  |  | **Weight** | 886 (17.2) | -2.71 | -4.33 | 1.33 | 0.98 - 1.80 | 0.068 |
|  |  | **BMI** | 826 (16) | -2.70 | -4.32 | 1.27 | 0.93 - 1.74 | 0.135 |
|  |  | **Residuals** | 848 (16.4) | -2.71 | -4.32 | 1.26 | 0.93 - 1.73 | 0.141 |

**Supplementary Table 7 - Association between sarcopenia and injurious falls in women using different methods of operationalizing sarcopenia**

| **Sarcopenia definition** | | | **Number of participants with sarcopenia (%)** | **Intercept 1** | **Intercept 2** | **Odds of injurious fall** | **95% Confidence interval** | **p-value** |
| --- | --- | --- | --- | --- | --- | --- | --- | --- |
| **Lean mass percentile** | **Combination of muscle variables** | **Method of adjusting lean mass** |  |  |  |  |  |  |
| **Not applicable** | **Grip strength <30kg** | | 1092 (22.9) | -2.27 | -3.87 | 1.01 | 0.79 - 1.29 | 0.948 |
|  | **Grip strength <26kg** | | 330 (6.9) | -2.27 | -3.87 | 0.89 | 0.60 - 1.34 | 0.584 |
|  | **Gait speed <0.8m/s** | | 1394 (29.2) | -2.28 | -3.89 | 1.14 | 0.90 - 1.43 | 0.279 |
|  | **Gait speed <1.0m/s** | | 3414 (71.5) | -2.23 | -3.83 | 0.93 | 0.74 - 1.18 | 0.543 |
|  | **Chair rise ≥15 seconds** | | 1977 (41.4) | -2.28 | -3.88 | 1.03 | 0.84 - 1.27 | 0.764 |
| **10th** | **Lean mass only** | **Height^2^** | 471 (9.9) | -2.28 | -3.88 | 1.11 | 0.79 - 1.54 | 0.547 |
|  |  | **Weight** | 468 (9.8) | -2.26 | -3.87 | 0.88 | 0.63 - 1.23 | 0.462 |
|  |  | **BMI** | 456 (9.6) | -2.26 | -3.86 | 0.78 | 0.55 - 1.11 | 0.174 |
|  |  | **Residuals** | 476 (10.0) | -2.26 | -3.86 | 0.88 | 0.63 - 1.24 | 0.465 |
|  | **Lean mass and grip strength <30kg** | **Height^2^** | 178 (3.7) | -2.26 | -3.87 | 0.81 | 0.46 - 1.43 | 0.467 |
|  |  | **Weight** | 165 (3.5) | -2.27 | -3.87 | 0.54 | 0.28 - 1.02 | 0.057 |
|  |  | **BMI** | 196 (4.1) | -2.27 | -3.87 | 0.38 | 0.20 - 0.73 | 0.004 |
|  |  | **Residuals** | 158 (3.3) | -2.27 | -3.87 | 0.72 | 0.39 - 1.31 | 0.280 |
|  | **Lean mass and grip strength <26kg** | **Height^2^** | 62 (1.3) | -2.27 | -3.87 | 0.51 | 0.17 - 1.52 | 0.229 |
|  |  | **Weight** | 48 (1.0) | -2.27 | -3.87 | 0.45 | 0.13 - 1.55 | 0.204 |
|  |  | **BMI** | 72 (1.5) | -2.27 | -3.87 | 0.47 | 0.18 - 1.24 | 0.127 |
|  |  | **Residuals** | 41 (0.9) | -2.27 | -3.87 | 0.78 | 0.26 - 2.33 | 0.653 |
|  | **Lean mass and gait speed <0.8m/s** | **Height^2^** | 131 (2.7) | -2.27 | -3.87 | 1.17 | 0.65 - 2.10 | 0.605 |
|  |  | **Weight** | 220 (4.6) | -2.27 | -3.87 | 1.03 | 0.66 - 1.61 | 0.901 |
|  |  | **BMI** | 215 (4.5) | -2.27 | -3.87 | 0.71 | 0.43 - 1.17 | 0.183 |
|  |  | **Residuals** | 168 (3.5) | -2.27 | -3.87 | 1.35 | 0.84 - 2.17 | 0.221 |
|  | **Lean mass and gait speed <1.0m/s** | **Height^2^** | 328 (6.9) | -2.27 | -3.88 | 1.08 | 0.73 - 1.59 | 0.712 |
|  |  | **Weight** | 399 (8.4) | -2.27 | -3.87 | 0.91 | 0.64 - 1.30 | 0.602 |
|  |  | **BMI** | 389 (8.1) | -2.26 | -3.86 | 0.72 | 0.49 - 1.06 | 0.098 |
|  |  | **Residuals** | 371 (7.8) | -2.26 | -3.87 | 0.91 | 0.63 - 1.32 | 0.624 |
|  | **Lean mass and chair rise ≥15 seconds** | **Height^2^** | 199 (4.2) | -2.28 | -3.88 | 1.20 | 0.74 - 1.95 | 0.450 |
|  |  | **Weight** | 221 (4.6) | -2.27 | -3.87 | 1.12 | 0.72 - 1.74 | 0.613 |
|  |  | **BMI** | 186 (3.9) | -2.27 | -3.87 | 0.76 | 0.45 - 1.28 | 0.298 |
|  |  | **Residuals** | 240 (5.0) | -2.27 | -3.87 | 1.02 | 0.65 - 1.58 | 0.945 |
| **20th** | **Lean mass only** | **Height^2^** | 960 (20.1) | -2.28 | -3.89 | 1.07 | 0.83 - 1.37 | 0.619 |
|  |  | **Weight** | 936 (19.6) | -2.26 | -3.86 | 0.93 | 0.72 - 1.19 | 0.551 |
|  |  | **BMI** | 930 (19.5) | -2.27 | -3.87 | 1.00 | 0.78 - 1.28 | 0.997 |
|  |  | **Residuals** | 950 (19.9) | -2.28 | -3.88 | 1.06 | 0.83 - 1.36 | 0.618 |
|  | **Lean mass and grip strength <30kg** | **Height^2^** | 329 (6.9) | -2.27 | -3.87 | 1.09 | 0.74 - 1.60 | 0.670 |
|  |  | **Weight** | 300 (6.3) | -2.27 | -3.87 | 0.56 | 0.35 - 0.90 | 0.016 |
|  |  | **BMI** | 361 (7.6) | -2.27 | -3.87 | 0.80 | 0.54 - 1.18 | 0.252 |
|  |  | **Residuals** | 280 (5.9) | -2.27 | -3.87 | 1.00 | 0.66 - 1.52 | 0.998 |
|  | **Lean mass and grip strength <26kg** | **Height^2^** | 118 (2.5) | -2.27 | -3.87 | 0.95 | 0.51 - 1.77 | 0.865 |
|  |  | **Weight** | 85 (1.8) | -2.27 | -3.87 | 0.48 | 0.20 - 1.20 | 0.116 |
|  |  | **BMI** | 121 (2.5) | -2.27 | -3.87 | 0.72 | 0.37 - 1.40 | 0.333 |
|  |  | **Residuals** | 86 (1.8) | -2.27 | -3.87 | 0.68 | 0.29 - 1.55 | 0.357 |
|  | **Lean mass and gait speed <0.8m/s** | **Height^2^** | 265 (5.6) | -2.28 | -3.88 | 1.29 | 0.86 - 1.94 | 0.220 |
|  |  | **Weight** | 409 (8.6) | -2.27 | -3.87 | 0.97 | 0.69 - 1.38 | 0.878 |
|  |  | **BMI** | 393 (8.2) | -2.27 | -3.87 | 1.09 | 0.77 - 1.54 | 0.629 |
|  |  | **Residuals** | 304 (6.4) | -2.28 | -3.88 | 1.46 | 1.01 - 2.10 | 0.043 |
|  | **Lean mass and gait speed <1.0m/s** | **Height^2^** | 686 (14.4) | -2.28 | -3.88 | 1.04 | 0.78 - 1.39 | 0.765 |
|  |  | **Weight** | 780 (16.3) | -2.26 | -3.86 | 0.91 | 0.69 - 1.19 | 0.498 |
|  |  | **BMI** | 766 (16.0) | -2.27 | -3.87 | 0.99 | 0.75 - 1.29 | 0.924 |
|  |  | **Residuals** | 715 (15.0) | -2.28 | -3.88 | 1.07 | 0.81 - 1.40 | 0.643 |
|  | **Lean mass and chair rise ≥15 seconds** | **Height^2^** | 406 (8.5) | -2.29 | -3.89 | 1.24 | 0.88 - 1.73 | 0.221 |
|  |  | **Weight** | 450 (9.4) | -2.27 | -3.87 | 0.95 | 0.68 - 1.33 | 0.781 |
|  |  | **BMI** | 411 (8.6) | -2.27 | -3.88 | 1.14 | 0.82 - 1.59 | 0.439 |
|  |  | **Residuals** | 450 (9.4) | -2.28 | -3.88 | 1.20 | 0.87 - 1.65 | 0.267 |
| **40th** | **Lean mass only** | **Height^2^** | 1912 (40.1) | -2.30 | -3.90 | 1.07 | 0.87 - 1.32 | 0.503 |
|  |  | **Weight** | 1900 (39.8) | -2.26 | -3.86 | 0.97 | 0.79 - 1.20 | 0.794 |
|  |  | **BMI** | 1882 (39.4) | -2.26 | -3.86 | 0.95 | 0.78 - 1.17 | 0.663 |
|  |  | **Residuals** | 1905 (39.9) | -2.31 | -3.91 | 1.10 | 0.90 - 1.35 | 0.341 |
|  | **Lean mass and grip strength <30kg** | **Height^2^** | 556 (11.6) | -2.28 | -3.88 | 1.11 | 0.82 - 1.52 | 0.494 |
|  |  | **Weight** | 531 (11.1) | -2.26 | -3.87 | 0.85 | 0.62 - 1.18 | 0.333 |
|  |  | **BMI** | 625 (13.1) | -2.26 | -3.86 | 0.77 | 0.56 - 1.06 | 0.104 |
|  |  | **Residuals** | 534 (11.2) | -2.27 | -3.87 | 0.96 | 0.69 - 1.32 | 0.783 |
|  | **Lean mass and grip strength <26kg** | **Height^2^** | 182 (3.8) | -2.27 | -3.87 | 1.20 | 0.74 - 1.95 | 0.462 |
|  |  | **Weight** | 150 (3.1) | -2.27 | -3.87 | 0.68 | 0.36 - 1.25 | 0.214 |
|  |  | **BMI** | 201 (4.2) | -2.27 | -3.87 | 0.64 | 0.37 - 1.11 | 0.111 |
|  |  | **Residuals** | 166 (3.5) | -2.27 | -3.87 | 0.94 | 0.55 - 1.62 | 0.829 |
|  | **Lean mass and gait speed <0.8m/s** | **Height^2^** | 490 (10.3) | -2.29 | -3.89 | 1.46 | 1.08 - 1.97 | 0.015 |
|  |  | **Weight** | 722 (15.1) | -2.27 | -3.87 | 1.01 | 0.76 - 1.33 | 0.944 |
|  |  | **BMI** | 747 (15.6) | -2.27 | -3.87 | 1.03 | 0.78 - 1.36 | 0.829 |
|  |  | **Residuals** | 581 (12.2) | -2.28 | -3.89 | 1.31 | 0.98 - 1.74 | 0.067 |
|  | **Lean mass and gait speed <1.0m/s** | **Height^2^** | 1303 (27.3) | -2.30 | -3.90 | 1.11 | 0.89 - 1.39 | 0.369 |
|  |  | **Weight** | 1502 (31.5) | -2.26 | -3.86 | 0.96 | 0.77 - 1.19 | 0.698 |
|  |  | **BMI** | 1499 (31.4) | -2.26 | -3.87 | 0.97 | 0.78 - 1.21 | 0.803 |
|  |  | **Residuals** | 1401 (29.3) | -2.29 | -3.89 | 1.10 | 0.88 - 1.36 | 0.410 |
|  | **Lean mass and chair rise ≥15 seconds** | **Height^2^** | 786 (16.5) | -2.29 | -3.89 | 1.15 | 0.88 - 1.49 | 0.296 |
|  |  | **Weight** | 868 (18.2) | -2.27 | -3.87 | 1.00 | 0.77 - 1.30 | 0.989 |
|  |  | **BMI** | 863 (18.1) | -2.26 | -3.87 | 0.93 | 0.71 - 1.20 | 0.562 |
|  |  | **Residuals** | 848 (17.8) | -2.29 | -3.89 | 1.14 | 0.89 - 1.47 | 0.301 |

**Supplementary Table 8 – Percentage of underweight, normal weight, overweight, and obese participants for each method of adjusting for lean mass using the 20^th^ percentile cut offs**

| **Men** | | | | | |
| --- | --- | --- | --- | --- | --- |
| **Body mass index** | **Number of participants in body mass index category (%)** | | | | |
|  | **ALM/height** | **ALM/weight** | **ALM/BMI** | **ALM Residuals** | **All participants** |
| **Underweight (<18.5kg/m2)** | 13 (1.3) | 0 (0) | 14 (1.4) | 9 (0.9) | 14 (0.3) |
| **Normal weight (18.5 - 24.9 kg/m2)** | 647 (63.7) | 52 (5.2) | 82 (8.2) | 396 (39.0) | 1274 (24.7) |
| **Overweight (25.0 - 29.9kg/m2)** | 322 (31.7) | 366 (36.8) | 411 (41.3) | 443 (43.6) | 2534 (49.1) |
| **Obese (≥30kg/m2)** | 34 (3.3) | 577 (58) | 501 (50.4) | 167 (16.5) | 1340 (26) |
| **Women** | | | | | |
|  | **Number of participants in body mass index category (%)** | | | | |
| **Body mass index** | **ALM/height** | **ALM/weight** | **ALM/BMI** | **ALM Residuals** | **All participants** |
| **Underweight (<18.5kg/m2)** | 52 (5.4) | 0 (0) | 0 (0) | 15 (1.6) | 63 (1.3) |
| **Normal weight (18.5 - 24.9 kg/m2)** | 719 (74.9) | 65 (6.9) | 113 (12.2) | 368 (38.7) | 1548 (32.4) |
| **Overweight (25.0 - 29.9kg/m2)** | 181 (18.9) | 291 (31.1) | 301 (32.4) | 369 (38.8) | 1788 (37.5) |
| **Obese (≥30kg/m2)** | 8 (0.8) | 580 (62.0) | 516 (55.5) | 198 (20.8) | 1375 (28.8) |

**Supplementary Table 9 – Number of men with zero, one, or two or more injurious falls stratified by sarcopenia status**

|  |  | **Sarcopenia No** | | | **Sarcopenia yes** | | | **1 or more falls** | | **2 or more falls** | |
| --- | --- | --- | --- | --- | --- | --- | --- | --- | --- | --- | --- |
|  |  | **0 Falls** | **1 Fall** | **2+ Falls** | **0 Falls** | **1 Fall** | **2+ Falls** | **Sensitivity** | **Specificity** | **Sensitivity** | **Specificity** |
| **Lean mass only (10th percentile)** | **ALM/height^2^** | 4239 | 334 | 89 | 444 | 41 | 15 | 0.12 | 0.91 | 0.14 | 1.00 |
|  | **ALM/weight** | 4257 | 321 | 91 | 426 | 54 | 13 | 0.14 | 0.91 | 0.13 | 1.00 |
|  | **ALM/BMI** | 4269 | 326 | 86 | 414 | 49 | 18 | 0.14 | 0.91 | 0.17 | 1.00 |
|  | **ALM regression** | 4242 | 327 | 86 | 441 | 48 | 18 | 0.14 | 0.91 | 0.17 | 1.00 |
| **Lean mass only (20th percentile)** | **ALM/height^2^** | 3778 | 288 | 80 | 905 | 87 | 24 | 0.23 | 0.81 | 0.23 | 0.99 |
|  | **ALM/weight** | 3818 | 272 | 77 | 865 | 103 | 27 | 0.27 | 0.82 | 0.26 | 0.99 |
|  | **ALM/BMI** | 3801 | 292 | 75 | 882 | 83 | 29 | 0.23 | 0.81 | 0.28 | 0.99 |
|  | **ALM regression** | 3791 | 277 | 79 | 892 | 98 | 25 | 0.26 | 0.81 | 0.24 | 0.99 |
| **Lean mass only (40th percentile)** | **ALM/height^2^** | 2851 | 210 | 57 | 1832 | 165 | 47 | 0.44 | 0.61 | 0.45 | 0.98 |
|  | **ALM/weight** | 2886 | 199 | 54 | 1797 | 176 | 50 | 0.47 | 0.62 | 0.48 | 0.98 |
|  | **ALM/BMI** | 2894 | 210 | 51 | 1789 | 165 | 53 | 0.46 | 0.62 | 0.51 | 0.98 |
|  | **ALM regression** | 2866 | 205 | 54 | 1817 | 170 | 50 | 0.46 | 0.61 | 0.48 | 0.98 |
| **Grip strength** | **(30kg and 20kg)** | 4110 | 307 | 77 | 573 | 68 | 27 | 0.20 | 0.88 | 0.26 | 0.99 |
| **Grip strength** | **(26kg and 16kg)** | 4471 | 342 | 92 | 212 | 33 | 12 | 0.09 | 0.95 | 0.12 | 1.00 |
| **Grip strength** | **(27kg and 16kg)** | 4407 | 331 | 91 | 276 | 44 | 13 | 0.12 | 0.94 | 0.13 | 1.00 |
| **Gait speed** | **1.0 mps** | 3688 | 274 | 62 | 995 | 101 | 42 | 0.30 | 0.79 | 0.40 | 0.99 |
| **Gait speed** | **0.80 mps** | 1657 | 126 | 29 | 3026 | 249 | 75 | 0.68 | 0.35 | 0.72 | 0.96 |
| **Chair rise** | **≥15 seconds** | 3019 | 231 | 53 | 1664 | 144 | 51 | 0.41 | 0.64 | 0.49 | 0.98 |
| **10th percentile - Lean mass and grip strength (30kg/20kg)** | **ALM/height^2^** | 4568 | 360 | 99 | 115 | 15 | 5 | 0.04 | 0.98 | 0.05 | 1.00 |
|  | **ALM/weight** | 4565 | 355 | 100 | 118 | 20 | 4 | 0.05 | 0.97 | 0.04 | 1.00 |
|  | **ALM/BMI** | 4532 | 357 | 95 | 151 | 18 | 9 | 0.06 | 0.97 | 0.09 | 1.00 |
|  | **ALM regression** | 4583 | 362 | 98 | 100 | 13 | 6 | 0.04 | 0.98 | 0.06 | 1.00 |
| **10th percentile - Lean mass and grip strength (26kg/16kg)** | **ALM/height^2^** | 4632 | 369 | 101 | 51 | 6 | 3 | 0.02 | 0.99 | 0.03 | 1.00 |
|  | **ALM/weight** | 4625 | 363 | 102 | 58 | 12 | 2 | 0.03 | 0.99 | 0.02 | 1.00 |
|  | **ALM/BMI** | 4616 | 366 | 98 | 67 | 9 | 6 | 0.03 | 0.99 | 0.06 | 1.00 |
|  | **ALM regression** | 4636 | 368 | 101 | 47 | 7 | 3 | 0.02 | 0.99 | 0.03 | 1.00 |
| **10th percentile - Lean mass and grip strength (27kg/16kg)** | **ALM/height^2^** | 4622 | 364 | 101 | 61 | 11 | 3 | 0.03 | 0.99 | 0.03 | 1.00 |
|  | **ALM/weight** | 4616 | 359 | 102 | 67 | 16 | 2 | 0.04 | 0.99 | 0.02 | 1.00 |
|  | **ALM/BMI** | 4604 | 362 | 98 | 79 | 13 | 6 | 0.04 | 0.98 | 0.06 | 1.00 |
|  | **ALM regression** | 4625 | 365 | 101 | 58 | 10 | 3 | 0.03 | 0.99 | 0.03 | 1.00 |
| **10th percentile - Lean mass and gait speed (0.8m/s)** | **ALM/height^2^** | 4555 | 362 | 97 | 128 | 13 | 7 | 0.04 | 0.97 | 0.07 | 1.00 |
|  | **ALM/weight** | 4512 | 352 | 98 | 171 | 23 | 6 | 0.06 | 0.96 | 0.06 | 1.00 |
|  | **ALM/BMI** | 4505 | 356 | 96 | 178 | 19 | 8 | 0.06 | 0.96 | 0.08 | 1.00 |
|  | **ALM regression** | 4553 | 355 | 96 | 130 | 20 | 8 | 0.06 | 0.97 | 0.08 | 1.00 |
| **10th percentile - Lean mass and gait speed (1.0m/s)** | **ALM/height^2^** | 4365 | 344 | 93 | 318 | 31 | 11 | 0.09 | 0.93 | 0.11 | 1.00 |
|  | **ALM/weight** | 4337 | 330 | 93 | 346 | 45 | 11 | 0.12 | 0.93 | 0.11 | 1.00 |
|  | **ALM/BMI** | 4342 | 335 | 90 | 341 | 40 | 14 | 0.11 | 0.93 | 0.13 | 1.00 |
|  | **ALM regression** | 4362 | 337 | 91 | 321 | 38 | 13 | 0.11 | 0.93 | 0.13 | 1.00 |
| **10th percentile - Lean mass and chair rise (≥15 seconds)** | **ALM/height^2^** | 4484 | 354 | 97 | 199 | 21 | 7 | 0.06 | 0.96 | 0.07 | 1.00 |
|  | **ALM/weight** | 4467 | 347 | 96 | 216 | 28 | 8 | 0.08 | 0.95 | 0.08 | 1.00 |
|  | **ALM/BMI** | 4488 | 352 | 91 | 195 | 23 | 13 | 0.08 | 0.96 | 0.13 | 1.00 |
|  | **ALM regression** | 4465 | 351 | 95 | 218 | 24 | 9 | 0.07 | 0.95 | 0.09 | 1.00 |
| **20th percentile - Lean mass and grip strength (30kg/20kg)** | **ALM/height^2^** | 4470 | 352 | 96 | 213 | 23 | 8 | 0.06 | 0.95 | 0.08 | 1.00 |
|  | **ALM/weight** | 4490 | 345 | 95 | 193 | 30 | 9 | 0.08 | 0.96 | 0.09 | 1.00 |
|  | **ALM/BMI** | 4435 | 343 | 90 | 248 | 32 | 14 | 0.10 | 0.95 | 0.13 | 1.00 |
|  | **ALM regression** | 4504 | 347 | 96 | 179 | 28 | 8 | 0.08 | 0.96 | 0.08 | 1.00 |
| **20th percentile - Lean mass and grip strength (26kg/16kg)** | **ALM/height^2^** | 4591 | 365 | 100 | 92 | 10 | 4 | 0.03 | 0.98 | 0.04 | 1.00 |
|  | **ALM/weight** | 4601 | 359 | 97 | 82 | 16 | 7 | 0.05 | 0.98 | 0.07 | 1.00 |
|  | **ALM/BMI** | 4585 | 358 | 96 | 98 | 17 | 8 | 0.05 | 0.98 | 0.08 | 1.00 |
|  | **ALM regression** | 4612 | 360 | 100 | 71 | 15 | 4 | 0.04 | 0.98 | 0.04 | 1.00 |
| **20th percentile - Lean mass and grip strength (27kg/16kg)** | **ALM/height^2^** | 4571 | 360 | 99 | 112 | 15 | 5 | 0.04 | 0.98 | 0.05 | 1.00 |
|  | **ALM/weight** | 4583 | 353 | 97 | 100 | 22 | 7 | 0.06 | 0.98 | 0.07 | 1.00 |
|  | **ALM/BMI** | 4562 | 352 | 96 | 121 | 23 | 8 | 0.06 | 0.97 | 0.08 | 1.00 |
|  | **ALM regression** | 4592 | 355 | 100 | 91 | 20 | 4 | 0.05 | 0.98 | 0.04 | 1.00 |
| **20th percentile - Lean mass and gait speed (0.8m/s)** | **ALM/height^2^** | 4462 | 351 | 95 | 221 | 24 | 9 | 0.07 | 0.95 | 0.09 | 1.00 |
|  | **ALM/weight** | 4385 | 338 | 91 | 298 | 37 | 13 | 0.10 | 0.94 | 0.13 | 1.00 |
|  | **ALM/BMI** | 4364 | 346 | 89 | 319 | 29 | 15 | 0.09 | 0.93 | 0.14 | 1.00 |
|  | **ALM regression** | 4446 | 343 | 93 | 237 | 32 | 11 | 0.09 | 0.95 | 0.11 | 1.00 |
| **20th percentile - Lean mass and gait speed (1.0m/s)** | **ALM/height^2^** | 4057 | 312 | 89 | 626 | 63 | 15 | 0.16 | 0.87 | 0.14 | 1.00 |
|  | **ALM/weight** | 4025 | 295 | 83 | 658 | 80 | 21 | 0.21 | 0.86 | 0.20 | 1.00 |
|  | **ALM/BMI** | 3989 | 309 | 79 | 694 | 66 | 25 | 0.19 | 0.85 | 0.24 | 0.99 |
|  | **ALM regression** | 4045 | 300 | 87 | 638 | 75 | 17 | 0.19 | 0.86 | 0.16 | 1.00 |
| **20th percentile - Lean mass and chair rise (≥15 seconds)** | **ALM/height^2^** | 4304 | 335 | 92 | 379 | 40 | 12 | 0.11 | 0.92 | 0.12 | 1.00 |
|  | **ALM/weight** | 4283 | 323 | 90 | 400 | 52 | 14 | 0.14 | 0.91 | 0.13 | 1.00 |
|  | **ALM/BMI** | 4320 | 336 | 85 | 363 | 39 | 19 | 0.12 | 0.92 | 0.18 | 1.00 |
|  | **ALM regression** | 4277 | 327 | 92 | 406 | 48 | 12 | 0.13 | 0.91 | 0.12 | 1.00 |
| **40th percentile - Lean mass and grip strength (30kg/20kg)** | **ALM/height^2^** | 4353 | 331 | 84 | 330 | 44 | 20 | 0.13 | 0.93 | 0.19 | 1.00 |
|  | **ALM/weight** | 4353 | 333 | 87 | 330 | 42 | 17 | 0.12 | 0.93 | 0.16 | 1.00 |
|  | **ALM/BMI** | 4311 | 328 | 84 | 372 | 47 | 20 | 0.14 | 0.92 | 0.19 | 1.00 |
|  | **ALM regression** | 4356 | 330 | 88 | 327 | 45 | 16 | 0.13 | 0.93 | 0.15 | 1.00 |
| **40th percentile - Lean mass and grip strength (26kg/16kg)** | **ALM/height^2^** | 4548 | 353 | 96 | 135 | 22 | 8 | 0.06 | 0.97 | 0.08 | 1.00 |
|  | **ALM/weight** | 4556 | 355 | 94 | 127 | 20 | 10 | 0.06 | 0.97 | 0.10 | 1.00 |
|  | **ALM/BMI** | 4543 | 350 | 94 | 140 | 25 | 10 | 0.07 | 0.97 | 0.10 | 1.00 |
|  | **ALM regression** | 4552 | 353 | 95 | 131 | 22 | 9 | 0.06 | 0.97 | 0.09 | 1.00 |
| **40th percentile - Lean mass and grip strength (27kg/16kg)** | **ALM/height^2^** | 4512 | 346 | 95 | 171 | 29 | 9 | 0.08 | 0.96 | 0.09 | 1.00 |
|  | **ALM/weight** | 4517 | 346 | 94 | 166 | 29 | 10 | 0.08 | 0.96 | 0.10 | 1.00 |
|  | **ALM/BMI** | 4500 | 340 | 94 | 183 | 35 | 10 | 0.09 | 0.96 | 0.10 | 1.00 |
|  | **ALM regression** | 4515 | 345 | 94 | 168 | 30 | 10 | 0.08 | 0.96 | 0.10 | 1.00 |
| **40th percentile - Lean mass and gait speed (0.8m/s)** | **ALM/height^2^** | 4255 | 329 | 87 | 428 | 46 | 17 | 0.13 | 0.91 | 0.16 | 1.00 |
|  | **ALM/weight** | 4165 | 320 | 77 | 518 | 55 | 27 | 0.17 | 0.89 | 0.26 | 0.99 |
|  | **ALM/BMI** | 4158 | 326 | 77 | 525 | 49 | 27 | 0.16 | 0.89 | 0.26 | 0.99 |
|  | **ALM regression** | 4222 | 322 | 82 | 461 | 53 | 22 | 0.16 | 0.90 | 0.21 | 1.00 |
| **40th percentile - Lean mass and gait speed (1.0m/s)** | **ALM/height^2^** | 3472 | 254 | 69 | 1211 | 121 | 35 | 0.33 | 0.74 | 0.34 | 0.99 |
|  | **ALM/weight** | 3385 | 246 | 64 | 1298 | 129 | 40 | 0.35 | 0.72 | 0.38 | 0.99 |
|  | **ALM/BMI** | 3366 | 252 | 60 | 1317 | 123 | 44 | 0.35 | 0.72 | 0.42 | 0.99 |
|  | **ALM regression** | 3438 | 248 | 64 | 1245 | 127 | 40 | 0.35 | 0.73 | 0.38 | 0.99 |
| **40th percentile - Lean mass and chair rise (≥15 seconds)** | **ALM/height^2^** | 3979 | 309 | 78 | 704 | 66 | 26 | 0.19 | 0.85 | 0.25 | 0.99 |
|  | **ALM/weight** | 3912 | 291 | 73 | 771 | 84 | 31 | 0.24 | 0.84 | 0.30 | 0.99 |
|  | **ALM/BMI** | 3964 | 303 | 69 | 719 | 72 | 35 | 0.22 | 0.85 | 0.34 | 0.99 |
|  | **ALM regression** | 3943 | 298 | 73 | 740 | 77 | 31 | 0.23 | 0.84 | 0.30 | 0.99 |

**Supplementary Table 10 – Number of women participants with zero, one, or two or more injurious falls stratified by sarcopenia status**

|  |  | **Sarcopenia No** | | | **Sarcopenia yes** | | | **1 or more falls** | | **2 or more falls** | |
| --- | --- | --- | --- | --- | --- | --- | --- | --- | --- | --- | --- |
|  |  | **0 Falls** | **1 Fall** | **2+ Falls** | **0 Falls** | **1 Fall** | **2+ Falls** | **Sensitivity** | **Specificity** | **Sensitivity** | **Specificity** |
| **Lean mass only (10th percentile)** | **ALM/height^2^** | 3756 | 421 | 126 | 405 | 47 | 19 | 0.11 | 0.90 | 0.13 | 1.00 |
|  | **ALM/weight** | 3753 | 427 | 126 | 408 | 41 | 19 | 0.10 | 0.90 | 0.13 | 1.00 |
|  | **ALM/BMI** | 3761 | 429 | 128 | 400 | 39 | 17 | 0.09 | 0.90 | 0.12 | 1.00 |
|  | **ALM regression** | 3744 | 427 | 127 | 417 | 41 | 18 | 0.10 | 0.90 | 0.12 | 1.00 |
| **Lean mass only (20th percentile)** | **ALM/height^2^** | 3329 | 375 | 110 | 832 | 93 | 35 | 0.21 | 0.80 | 0.24 | 0.99 |
|  | **ALM/weight** | 3351 | 384 | 103 | 810 | 84 | 42 | 0.21 | 0.81 | 0.29 | 0.99 |
|  | **ALM/BMI** | 3362 | 382 | 100 | 799 | 86 | 45 | 0.21 | 0.81 | 0.31 | 0.99 |
|  | **ALM regression** | 3342 | 374 | 108 | 819 | 94 | 37 | 0.21 | 0.80 | 0.26 | 0.99 |
| **Lean mass only (40th percentile)** | **ALM/height^2^** | 2498 | 279 | 85 | 1663 | 189 | 60 | 0.41 | 0.60 | 0.41 | 0.98 |
|  | **ALM/weight** | 2522 | 271 | 81 | 1639 | 197 | 64 | 0.43 | 0.61 | 0.44 | 0.98 |
|  | **ALM/BMI** | 2540 | 278 | 74 | 1621 | 190 | 71 | 0.43 | 0.61 | 0.49 | 0.98 |
|  | **ALM regression** | 2514 | 273 | 82 | 1647 | 195 | 63 | 0.42 | 0.60 | 0.43 | 0.98 |
| **Grip strength** | **(30kg and 20kg)** | 3224 | 355 | 103 | 937 | 113 | 42 | 0.25 | 0.77 | 0.29 | 0.99 |
| **Grip strength** | **(26kg and 16kg)** | 3875 | 434 | 135 | 286 | 34 | 10 | 0.07 | 0.93 | 0.07 | 1.00 |
| **Gait speed** | **1.0 mps** | 2986 | 318 | 76 | 1175 | 150 | 69 | 0.36 | 0.72 | 0.48 | 0.98 |
| **Gait speed** | **0.80 mps** | 1204 | 125 | 31 | 2957 | 343 | 114 | 0.75 | 0.29 | 0.79 | 0.92 |
| **Chair rise** | **≥15 seconds** | 2461 | 271 | 65 | 1700 | 197 | 80 | 0.45 | 0.59 | 0.55 | 0.97 |
| **10th percentile - Lean mass and grip strength (30kg/20kg)** | **ALM/height^2^** | 4005 | 452 | 139 | 156 | 16 | 6 | 0.04 | 0.96 | 0.04 | 1.00 |
|  | **ALM/weight** | 4013 | 457 | 139 | 148 | 11 | 6 | 0.03 | 0.96 | 0.04 | 1.00 |
|  | **ALM/BMI** | 3981 | 458 | 139 | 180 | 10 | 6 | 0.03 | 0.96 | 0.04 | 1.00 |
|  | **ALM regression** | 4022 | 457 | 137 | 139 | 11 | 8 | 0.03 | 0.97 | 0.06 | 1.00 |
| **10th percentile - Lean mass and grip strength (26kg/16kg)** | **ALM/height^2^** | 4105 | 464 | 143 | 56 | 4 | 2 | 0.01 | 0.99 | 0.01 | 1.00 |
|  | **ALM/weight** | 4117 | 464 | 145 | 44 | 4 | 0 | 0.01 | 0.99 | 0.00 | 1.00 |
|  | **ALM/BMI** | 4095 | 464 | 143 | 66 | 4 | 2 | 0.01 | 0.98 | 0.01 | 1.00 |
|  | **ALM regression** | 4125 | 464 | 144 | 36 | 4 | 1 | 0.01 | 0.99 | 0.01 | 1.00 |
| **10th percentile - Lean mass and gait speed (0.8m/s)** | **ALM/height^2^** | 4053 | 457 | 133 | 108 | 11 | 12 | 0.04 | 0.97 | 0.08 | 1.00 |
|  | **ALM/weight** | 3972 | 450 | 132 | 189 | 18 | 13 | 0.05 | 0.95 | 0.09 | 1.00 |
|  | **ALM/BMI** | 3972 | 453 | 134 | 189 | 15 | 11 | 0.04 | 0.95 | 0.08 | 1.00 |
|  | **ALM regression** | 4023 | 452 | 131 | 138 | 16 | 14 | 0.05 | 0.97 | 0.10 | 1.00 |
| **10th percentile - Lean mass and gait speed (1.0m/s)** | **ALM/height^2^** | 3882 | 436 | 128 | 279 | 32 | 17 | 0.08 | 0.93 | 0.12 | 1.00 |
|  | **ALM/weight** | 3815 | 432 | 128 | 346 | 36 | 17 | 0.09 | 0.92 | 0.12 | 1.00 |
|  | **ALM/BMI** | 3818 | 437 | 130 | 343 | 31 | 15 | 0.08 | 0.92 | 0.10 | 1.00 |
|  | **ALM regression** | 3839 | 436 | 128 | 322 | 32 | 17 | 0.08 | 0.92 | 0.12 | 1.00 |
| **10th percentile - Lean mass and chair rise (≥15 seconds)** | **ALM/height^2^** | 3994 | 450 | 131 | 167 | 18 | 14 | 0.05 | 0.96 | 0.10 | 1.00 |
|  | **ALM/weight** | 3976 | 446 | 131 | 185 | 22 | 14 | 0.06 | 0.96 | 0.10 | 1.00 |
|  | **ALM/BMI** | 4000 | 453 | 135 | 161 | 15 | 10 | 0.04 | 0.96 | 0.07 | 1.00 |
|  | **ALM regression** | 3956 | 448 | 130 | 205 | 20 | 15 | 0.06 | 0.95 | 0.10 | 1.00 |
| **20th percentile - Lean mass and grip strength (30kg/20kg)** | **ALM/height^2^** | 3880 | 434 | 131 | 281 | 34 | 14 | 0.08 | 0.93 | 0.10 | 1.00 |
|  | **ALM/weight** | 3894 | 447 | 133 | 267 | 21 | 12 | 0.05 | 0.94 | 0.08 | 1.00 |
|  | **ALM/BMI** | 3848 | 437 | 128 | 313 | 31 | 17 | 0.08 | 0.92 | 0.12 | 1.00 |
|  | **ALM regression** | 3925 | 438 | 131 | 236 | 30 | 14 | 0.07 | 0.94 | 0.10 | 1.00 |
| **20th percentile - Lean mass and grip strength (26kg/16kg)** | **ALM/height^2^** | 4059 | 455 | 142 | 102 | 13 | 3 | 0.03 | 0.98 | 0.02 | 1.00 |
|  | **ALM/weight** | 4083 | 462 | 144 | 78 | 6 | 1 | 0.01 | 0.98 | 0.01 | 1.00 |
|  | **ALM/BMI** | 4055 | 457 | 141 | 106 | 11 | 4 | 0.02 | 0.97 | 0.03 | 1.00 |
|  | **ALM regression** | 4085 | 460 | 143 | 76 | 8 | 2 | 0.02 | 0.98 | 0.01 | 1.00 |
| **20th percentile - Lean mass and gait speed (0.8m/s)** | **ALM/height^2^** | 3942 | 442 | 125 | 219 | 26 | 20 | 0.08 | 0.95 | 0.14 | 1.00 |
|  | **ALM/weight** | 3812 | 435 | 118 | 349 | 33 | 27 | 0.10 | 0.92 | 0.19 | 0.99 |
|  | **ALM/BMI** | 3830 | 433 | 118 | 331 | 35 | 27 | 0.10 | 0.92 | 0.19 | 0.99 |
|  | **ALM regression** | 3914 | 434 | 122 | 247 | 34 | 23 | 0.09 | 0.94 | 0.16 | 0.99 |
| **20th percentile - Lean mass and gait speed (1.0m/s)** | **ALM/height^2^** | 3571 | 403 | 114 | 590 | 65 | 31 | 0.16 | 0.86 | 0.21 | 0.99 |
|  | **ALM/weight** | 3487 | 398 | 109 | 674 | 70 | 36 | 0.17 | 0.84 | 0.25 | 0.99 |
|  | **ALM/BMI** | 3505 | 396 | 107 | 656 | 72 | 38 | 0.18 | 0.84 | 0.26 | 0.99 |
|  | **ALM regression** | 3550 | 395 | 114 | 611 | 73 | 31 | 0.17 | 0.85 | 0.21 | 0.99 |
| **20th percentile - Lean mass and chair rise (≥15 seconds)** | **ALM/height^2^** | 3820 | 428 | 120 | 341 | 40 | 25 | 0.11 | 0.92 | 0.17 | 0.99 |
|  | **ALM/weight** | 3777 | 429 | 118 | 384 | 39 | 27 | 0.11 | 0.91 | 0.19 | 0.99 |
|  | **ALM/BMI** | 3820 | 426 | 117 | 341 | 42 | 28 | 0.11 | 0.92 | 0.19 | 0.99 |
|  | **ALM regression** | 3783 | 423 | 118 | 378 | 45 | 27 | 0.12 | 0.91 | 0.19 | 0.99 |
| **40th percentile - Lean mass and grip strength (30kg/20kg)** | **ALM/height^2^** | 3687 | 408 | 123 | 474 | 60 | 22 | 0.13 | 0.89 | 0.15 | 0.99 |
|  | **ALM/weight** | 3705 | 415 | 123 | 456 | 53 | 22 | 0.12 | 0.89 | 0.15 | 0.99 |
|  | **ALM/BMI** | 3620 | 409 | 120 | 541 | 59 | 25 | 0.14 | 0.87 | 0.17 | 0.99 |
|  | **ALM regression** | 3703 | 414 | 123 | 458 | 54 | 22 | 0.12 | 0.89 | 0.15 | 0.99 |
| **40th percentile - Lean mass and grip strength (26kg/16kg)** | **ALM/height^2^** | 4009 | 445 | 138 | 152 | 23 | 7 | 0.05 | 0.96 | 0.05 | 1.00 |
|  | **ALM/weight** | 4029 | 454 | 141 | 132 | 14 | 4 | 0.03 | 0.97 | 0.03 | 1.00 |
|  | **ALM/BMI** | 3983 | 449 | 141 | 178 | 19 | 4 | 0.04 | 0.96 | 0.03 | 1.00 |
|  | **ALM regression** | 4018 | 451 | 139 | 143 | 17 | 6 | 0.04 | 0.97 | 0.04 | 1.00 |
| **40th percentile - Lean mass and gait speed (0.8m/s)** | **ALM/height^2^** | 3762 | 408 | 114 | 399 | 60 | 31 | 0.15 | 0.90 | 0.21 | 0.99 |
|  | **ALM/weight** | 3550 | 395 | 107 | 611 | 73 | 38 | 0.18 | 0.85 | 0.26 | 0.99 |
|  | **ALM/BMI** | 3533 | 393 | 101 | 628 | 75 | 44 | 0.19 | 0.85 | 0.30 | 0.99 |
|  | **ALM regression** | 3679 | 405 | 109 | 482 | 63 | 36 | 0.16 | 0.88 | 0.25 | 0.99 |
| **40th percentile - Lean mass and gait speed (1.0m/s)** | **ALM/height^2^** | 3043 | 330 | 98 | 1118 | 138 | 47 | 0.30 | 0.73 | 0.32 | 0.99 |
|  | **ALM/weight** | 2871 | 311 | 90 | 1290 | 157 | 55 | 0.35 | 0.69 | 0.38 | 0.98 |
|  | **ALM/BMI** | 2879 | 312 | 84 | 1282 | 156 | 61 | 0.35 | 0.69 | 0.42 | 0.98 |
|  | **ALM regression** | 2960 | 321 | 92 | 1201 | 147 | 53 | 0.33 | 0.71 | 0.37 | 0.98 |
| **40th percentile - Lean mass and chair rise (≥15 seconds)** | **ALM/height^2^** | 3490 | 388 | 110 | 671 | 80 | 35 | 0.19 | 0.84 | 0.24 | 0.99 |
|  | **ALM/weight** | 3425 | 378 | 103 | 736 | 90 | 42 | 0.22 | 0.82 | 0.29 | 0.99 |
|  | **ALM/BMI** | 3423 | 388 | 100 | 738 | 80 | 45 | 0.20 | 0.82 | 0.31 | 0.99 |
|  | **ALM regression** | 3440 | 382 | 104 | 721 | 86 | 41 | 0.21 | 0.83 | 0.28 | 0.99 |

**Supplemetnary Table 11 – Association between sarcopenia and injurious falls in European men using different methods of operationalizing sarcopenia**

| **Sarcopenia definition** | | **Number of participants with sarcopenia (%)** | **Y-intercept for 1+ injurious falls versus no falls** | **Y-intercept for 2+ injurious falls versus zero or one falls** | **Odds of falling** | **95% Confidence interval** | **p-value** |
| --- | --- | --- | --- | --- | --- | --- | --- |
|  |  |  |  |  |  |  |  |
| **Combination of muscle variables** | **Method of adjusting lean mass** |  |  |  |  |  |  |
| **Lean mass only (20th percentile)** | **Height**^2^ | 953 (19.2) | -2.69 | -4.32 | 1.21 | 0.89 - 1.64 | 0.222 |
|  | **Weight** | 956 (19.3) | -2.70 | -4.33 | 1.40 | 1.05 - 1.88 | 0.024* |
|  | **BMI** | 939 (19.0) | -2.67 | -4.29 | 1.06 | 0.78 - 1.44 | 0.718 |
|  | **Residuals** | 973 (19.7) | -2.71 | -4.33 | 1.33 | 0.99 - 1.78 | 0.060 |
| **Grip strength only <30kg** | | 617 (12.5) | -2.68 | -4.31 | 1.49 | 1.06 - 2.09 | 0.023* |
| **Grip strength only <26kg** | | 236 (4.8) | -2.67 | -4.29 | 1.83 | 1.14 - 2.94 | 0.013* |
| **Gait speed only <0.8m/s** | | 1076 (21.7) | -2.69 | -4.31 | 1.29 | 0.96 - 1.73 | 0.093 |
| **Lean mass (20th percentile) and grip strength <30kg** | **Height**^2^ | 225 (4.5) | -2.66 | -4.29 | 1.25 | 0.73 - 2.16 | 0.415 |
|  | **Weight** | 219 (4.4) | -2.66 | -4.29 | 1.68 | 1.02 - 2.76 | 0.041* |
|  | **BMI** | 268 (5.4) | -2.67 | -4.29 | 1.63 | 1.03 - 2.57 | 0.038* |
|  | **Residuals** | 201 (4.1) | -2.67 | -4.29 | 1.80 | 1.08 - 2.99 | 0.024* |
| **Lean mass (20th percentile) and grip strength <26kg** | **Height**^2^ | 97 (2.0) | -2.66 | -4.28 | 1.29 | 0.58 - 2.86 | 0.525 |
|  | **Weight** | 99 (2.0) | -2.66 | -4.29 | 2.17 | 1.11 - 4.24 | 0.024* |
|  | **BMI** | 112 (2.3) | -2.67 | -4.29 | 2.06 | 1.09 - 3.89 | 0.026* |
|  | **Residuals** | 84 (1.7) | -2.66 | -4.28 | 2.24 | 1.11 - 4.52 | 0.025* |
| **Lean mass (20th percentile) and gait speed <0.8m/s** | **Height**^2^ | 237 (4.8) | -2.66 | -4.28 | 1.11 | 0.65 - 1.92 | 0.699 |
|  | **Weight** | 326 (6.6) | -2.66 | -4.29 | 1.19 | 0.76 - 1.88 | 0.443 |
|  | **BMI** | 338 (6.8) | -2.66 | -4.28 | 0.97 | 0.61 - 1.55 | 0.899 |
|  | **Residuals** | 267 (5.4) | -2.67 | -4.29 | 1.30 | 0.80 - 2.12 | 0.286 |

* Statistically significant (p<0.05)

**Supplementary Table 12 – Association between sarcopenia and falls in European women using different methods of operationalizing sarcopenia**

| **Sarcopenia definition** | | **Number of participants with sarcopenia (%)** | **Y-intercept for 1+ injurious falls versus no falls** | **Y-intercept for 2+ injurious falls versus zero or one falls** | **Odds of falling** | **95% Confidence interval** | **p-value** |
| --- | --- | --- | --- | --- | --- | --- | --- |
|  |  |  |  |  |  |  |  |
| **Combination of muscle variables** | **Method of adjusting lean mass** |  |  |  |  |  |  |
| **Lean mass only (20th percentile)** | **Height**^2^ | 928 (20.0) | -2.29 | -3.87 | 1.09 | 0.84 - 1.40 | 0.510 |
|  | **Weight** | 913 (19.6) | -2.25 | -3.87 | 0.89 | 0.69 - 1.15 | 0.387 |
|  | **BMI** | 899 (19.3) | -2.27 | -3.87 | 1.02 | 0.79 - 1.31 | 0.896 |
|  | **Residuals** | 930 (20.0) | -2.28 | -3.87 | 1.05 | 0.82 - 1.35 | 0.688 |
| **Grip strength only <20kg** | | 1056 (22.7) | -2.27 | -3.87 | 1.01 | 0.79 - 1.30 | 0.927 |
| **Grip strength only <16kg** | | 314 (6.8) | -2.26 | -3.87 | 0.89 | 0.59 - 1.35 | 0.586 |
| **Gait speed only <0.8m/s** | | 1335 (28.7) | -2.28 | -3.89 | 1.16 | 0.92 - 1.46 | 0.224 |
| **Lean mass (20th percentile) and grip strength <20kg** | **Height**^2^ | 315 (6.8) | -2.27 | -3.87 | 1.09 | 0.73 - 1.62 | 0.679 |
|  | **Weight** | 289 (6.2) | -2.26 | -3.87 | 0.53 | 0.32 - 0.86 | 0.010* |
|  | **BMI** | 344 (7.4) | -2.26 | -3.87 | 0.81 | 0.54 - 1.21 | 0.301 |
|  | **Residuals** | 272 (5.8) | -2.27 | -3.87 | 0.99 | 0.64 - 1.51 | 0.955 |
| **Lean mass (20th percentile) and grip strength <16kg** | **Height**^2^ | 111 (2.4) | -2.27 | -3.87 | 0.98 | 0.52 - 1.86 | 0.959 |
|  | **Weight** | 79 (1.7) | -2.26 | -3.87 | 0.40 | 0.15 - 1.10 | 0.077 |
|  | **BMI** | 111 (2.4) | -2.27 | -3.87 | 0.70 | 0.35 - 1.42 | 0.325 |
|  | **Residuals** | 82 (1.8) | -2.26 | -3.87 | 0.67 | 0.28 - 1.58 | 0.357 |
| **Lean mass (20th percentile) and gait speed <0.8m/s** | **Height**^2^ | 252 (5.4) | -2.28 | -3.88 | 1.36 | 0.90 - 2.06 | 0.140 |
|  | **Weight** | 396 (8.5) | -2.27 | -3.87 | 0.94 | 0.66 - 1.35 | 0.750 |
|  | **BMI** | 374 (8.0) | -2.27 | -3.87 | 1.12 | 0.79 - 1.59 | 0.531 |
|  | **Residuals** | 296 (6.4) | -2.27 | -3.88 | 1.43 | 0.99 - 2.08 | 0.057 |

* Statistically significant (p<0.05)
